# The relevance of the superior cervical ganglion for cardiac autonomic innervation in health and disease: a systematic review

**DOI:** 10.1007/s10286-024-01019-2

**Published:** 2024-02-23

**Authors:** H. Sophia Chen, Lieke van Roon, Yang Ge, Janine M. van Gils, Jan W. Schoones, Marco C. DeRuiter, Katja Zeppenfeld, Monique R. M. Jongbloed

**Affiliations:** 1 Department of Cardiology, Willem Einthoven Center for Cardiac Arrhythmia Research and Management, Leiden University Medical Center, Leiden, The Netherlands; 2 Department of Anatomy & Embryology, Leiden University Medical Center, Leiden, The Netherlands; 3 Directorate of Research Policy, Leiden University Medical Center, Leiden, The Netherlands; 4 Department of Cardiology, Center of Congenital Heart Disease Amsterdam Leiden (CAHAL), Leiden University Medical Center, Leiden, The Netherlands

**Keywords:** Autonomic nervous system, Cardiac innervation, Superior cervical ganglion, Sympathetic ganglia, Sympathetic chain

## Abstract

**Purpose:**

The heart receives cervical and thoracic sympathetic contributions. Although the stellate ganglion is considered the main contributor to cardiac sympathetic innervation, the superior cervical ganglia (SCG) is used in many experimental studies. The clinical relevance of the SCG to cardiac innervation is controversial. We investigated current morphological and functional evidence as well as controversies on the contribution of the SCG to cardiac innervation.

**Methods:**

A systematic literature review was conducted in PubMed, Embase, Web of Science, and COCHRANE Library. Included studies received a full/text review and quality appraisal.

**Results:**

Seventy-six eligible studies performed between 1976 and 2023 were identified. In all species studied, morphological evidence of direct or indirect SCG contribution to cardiac innervation was found, but its contribution was limited. Morphologically, SCG sidedness may be relevant. There is indirect functional evidence that the SCG contributes to cardiac innervation as shown by its involvement in sympathetic overdrive reactions in cardiac disease states. A direct functional contribution was not found. Functional data on SCG sidedness was largely unavailable. Information about sex differences and pre- and postnatal differences was lacking.

**Conclusion:**

Current literature mainly supports an indirect involvement of the SCG in cardiac innervation, via other structures and plexuses or via sympathetic overdrive in response to cardiac diseases. Morphological evidence of a direct involvement was found, but its contribution seems limited. The relevance of SCG sidedness, sex, and developmental stage in health and disease remains unclear and warrants further exploration.

**Graphical abstract:**

An overview of the current literature derived from morphological and functional data on the involvement of SCG in cardiac innervation, relevance of sidedness, sex differences, and pre- and postnatal differences in various species. X = Information not available

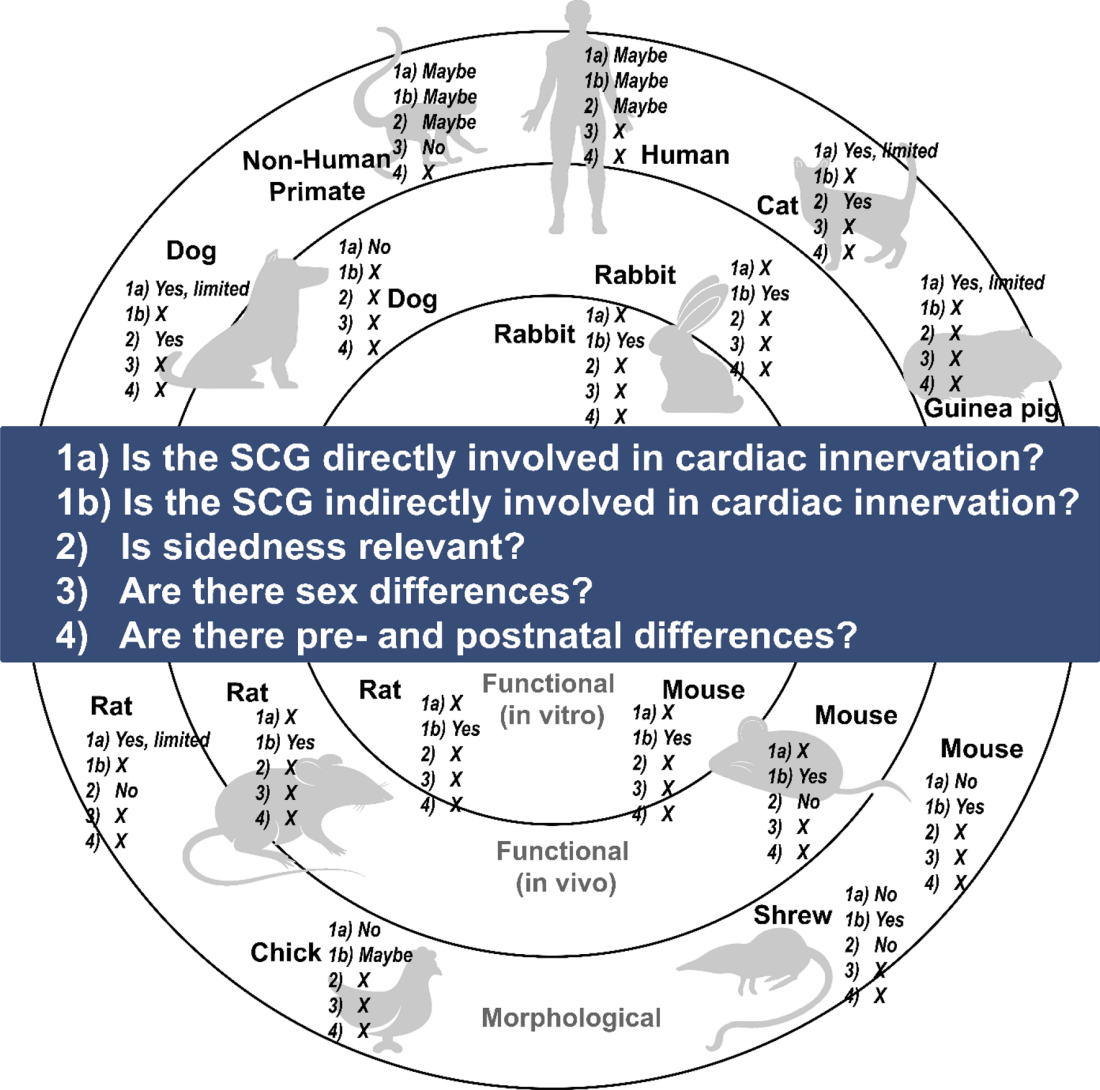

**Supplementary Information:**

The online version contains supplementary material available at 10.1007/s10286-024-01019-2.

## Introduction

A balanced function of the cardiac autonomic nervous system is essential to maintain cardiovascular homeostasis. Cardiac innervation is provided by the autonomic nervous system, which is organized into sympathetic and parasympathetic branches. Balancing sympathetic and parasympathetic tone is mandatory to maintain a regular heartbeat. Parasympathetic innervation of the heart is provided by preganglionic branches of the vagal nerve that synapse close to the target organ, e.g., in intrinsic cardiac ganglia situated in the myocardial wall and on the epicardial surface of the heart [1]. For sympathetic innervation, preganglionic sympathetic axons synapse with sympathetic neurons in the sympathetic chain, after which postganglionic fibers directly innervate either the myocardium or first synapse on the intrinsic cardiac ganglia [1]. In humans, this innervation from the sympathetic chain is likely provided by both cervical and thoracic ganglia, although the exact level of ganglia contributing to the heart is still controversial [2].

Interest in cardiac autonomic innervation has increased in the past decades, as alterations in cardiac innervation, both morphologically and functionally, after cardiac damage have been reported in myriad studies [3–6]. An especially intriguing phenomenon is the so-called cardiac sympathetic hyperinnervation, which can occur after cardiac damage, such as myocardial infarction (MI) [7, 8]. This hyperinnervation is characterized by an increased amount of sympathetic nerve fibers in the area of damage and has been related to ventricular arrhythmias and sudden cardiac death after MI [6]. Although several excellent mechanistic studies have been performed, the exact underlying relation between the occurrence of sympathetic hyperinnervation and ventricular arrhythmias after MI is still being determined. Apparently sympathetic ganglia, renowned for their limited growth potential after birth, retrieve their potential for fast outgrowth after cardiac damage. These findings have prompted researchers to study cardiac innervation in health and disease.

**Fig. 1:**
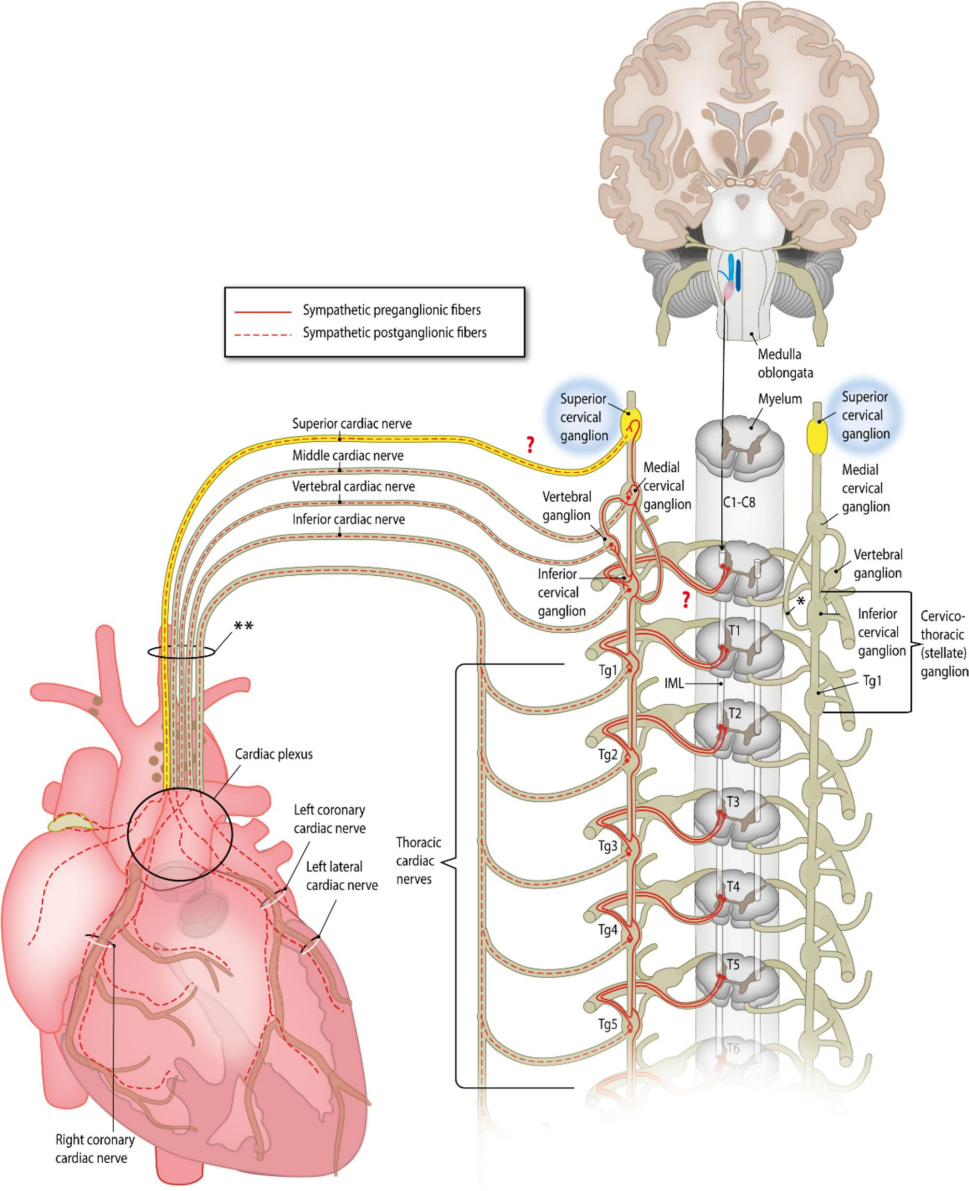
Anatomy of the cardiac sympathetic nervous system. Schematic drawing of the cardiac sympathetic nervous system. Preganglionic sympathetic axons from spinal cord neurons synapse with postganglionic sympathetic neurons in the ganglia of the sympathetic chain, running bilaterally along the vertebral column. Postganglionic fibers from these ganglia form the sympathetic cardiac nerves, which join in the cardiac plexus together with the parasympathetic nerves, providing the autonomic innervation of the heart. The superior cervical ganglia are indicated in bright yellow. The superior cardiac nerve, the existence of which is disputed in some studies, is shown in bright yellow. Figure adapted from “Human adult cardiac autonomic innervation: controversies in anatomical knowledge and relevance for cardiac neuromodulation,” by Wink et al. *Autonomic Neuroscience*, 2020. 227: p. 102,674. Copyright 2020 by Copyright Clearance Center

The stellate ganglia, consisting of the fused inferior cervical ganglion with the first thoracic ganglion, are most renowned for their contribution to cardiac innervation. They are located deeper in the thorax anterior to the first rib (Fig. 1). Although the stellate ganglion is generally accepted to provide a majority of cardiac innervation, other ganglia have been proposed to contribute to health and disease as well, including the thoracic and cervical ganglia [2, 9]. Cervical ganglia have indeed been shown to contribute to cardiac innervation both in animal models as well as in human, however reports in literature differ [2]. These ganglia also contribute to innervation of other structures in the head and neck, including the iris, jaw submandibular gland, the pineal gland, and the carotid body [10–12]. One of the cervical ganglia is the superior cervical ganglion (SCG) (Fig. 1), which is in close spatial orientation with the carotid body, a chemoreceptor-sensitive organ that can respond to changes in blood oxygen, carbon dioxide, and pH levels, as well as with the ganglion nodosum, the inferior ganglion of the vagal nerve [13, 14]. From a basic science point of view, the relatively good accessibility of the SCG, which is located at a specific anatomical landmark location, at the bifurcation of the common carotid arteries, provides advantages when using this structure as an experimental model to study cardiac (hyper)innervation. A prerequisite for using the SCG to study cardiac autonomic function is that this structure provides a significant anatomical and/or functional contribution to cardiac innervation.

Consistent differences have been shown in cardiac autonomic regulation between women and men, such as a more pronounced parasympathetic cardiac regulation, higher resting heart rate, and lower baroflex sensitivity in women, although its physiologic usefulness remains largely unknown [15]. Another factor that could possible influence the anatomical and functional contribution to cardiac innervation is the sidedness of the ganglia. The human peripheral cardiac autonomic nervous system shows considerable asymmetry, interindividual variations and regional differences in anatomical, functional, and molecular characteristics [16]. Moreover, MI has been shown to induce morphologic and neurochemical changes in right- and left-sided ganglia [5, 17].

In this review, we aim to systematically investigate current morphological and functional evidence, as well as to expose current controversies and gaps in knowledge, on the contribution of the SCG to cardiac innervation in health and disease in human and other animal models, including the consideration of potentially relevant aspects such as sex and sidedness.

## Methods

### Research questions

To perform a comprehensive systematic review of current knowledge on the contribution of the SCG in cardiac autonomic innervation, our research questions were defined as follows:

1. What is the morphological and functional evidence that the SCG is involved in cardiac innervation in various species, in health as well as in cardiac disease?
  - Is sidedness relevant (e.g., using left- or right-sided SCG) to study cardiac innervation?
  - Have sex differences been studied and/or encountered?
  - Are there pre- and postnatal/adult differences?
  - What is the quality of the included studies?
2. Which controversies are encountered, and which questions are potentially unanswered by current data?
3. And finally, derived from these data: Is the use of the SCG in experimental setting an adequate structure to study cardiac innervation in health and disease?

### Search strategy

This systematic review was conducted in PubMed, Embase, Web of Science, and COCHRANE Library databases up to 4 April 2023 and was in adherence with the Preferred Reporting Items for Systematic Reviews and Meta-Analyses (PRISMA) statement guidelines [18], using the same workflow as in previously published systematic reviews [2, 19, 20]. The search strategy was conducted by using keywords for superior cervical ganglion, heart, innervation, and nerve growth factor. The full search strategy for each respective database can be viewed in the Supplementary Materials (Appendix A).

### Selection criteria

Papers were considered eligible to be included in this systematic review when both the SCG and the heart were studied. Both studies in health and in disease models were included. Additionally, all animal species including humans, both prenatal and postnatal/adult studies, were included. Functional studies, in vivo as well as in vitro, were included as long as an interdependent effect of the SCG on the heart or vice versa was studied. Papers in which the presence of a direct/causal link between the SCG and the heart was not studied were excluded, e.g., when the effect of certain substances was studied separately on the heart and SCG or when immunostaining was performed on the SCG and/or heart without an intervention to the opposing tissue. Only original research papers were included; reviews, editorials, and book chapters were excluded. Other reasons to exclude the paper were: non-English-language papers, papers that could not be retrieved after significant effort, and papers that were published in a predatory journal [21]. With regard to the date of publication, articles published before 1975 were excluded, partly owing to the difficulties in retrieving several older papers. The reference lists of included papers were searched for eligible articles that were not identified with the query.

### Data extraction and quality appraisal

All records were screened for eligibility by two independent authors (H.S.C. and M.R.M.J.) on the basis of titles and abstracts followed by full-text review where necessary, considering the selection criteria described above.

Selected papers were categorized into (1) morphological, (2) functional in vivo, or (3) functional in vitro. In case multiple categories were applicable, the paper was included in all categories and only data relating to the category were extracted and assessed for quality. Anterograde or retrograde labeling studies were considered as morphological studies.

When available, the following data were extracted independently by two authors (H.S.C. and L.V.R.): author and year of publication; objectives; number; species; strain/breed; genotype; age; weight; sex; study type; data on sidedness; experimental condition; experimental setting; solution and staining or histochemistry; SCG-heart-related outcome; limitations. Discrepancies between the observers’ judgments were resolved by discussion and consensus.

The methodological quality of the morphological studies was scored by two authors (H.S.C. and M.R.M.J.) using the Quality Appraisal for Cadaveric Studies (QUACS) scale [22]. Similarly, the methodological quality of the functional studies was scored by two authors (H.S.C. and L.V.R.) using the Animal Research: Reporting In Vivo Experiments (ARRIVE) guidelines 2.0 [23] for the in vivo studies and an adapted version for the in vitro studies (Supplementary Materials, Appendix B) as no existing quality assessment tool currently covers all critical aspects of in vitro studies [24].

After comparing and discussing the individual scores for each paper, a consensus score was achieved for all papers included in this review.

Note that all extracted data and quality assessments were solely based on aspects concerning the relationship between the SCG and the heart.

## Results

### Study selection and inclusion

**Fig. 2:**
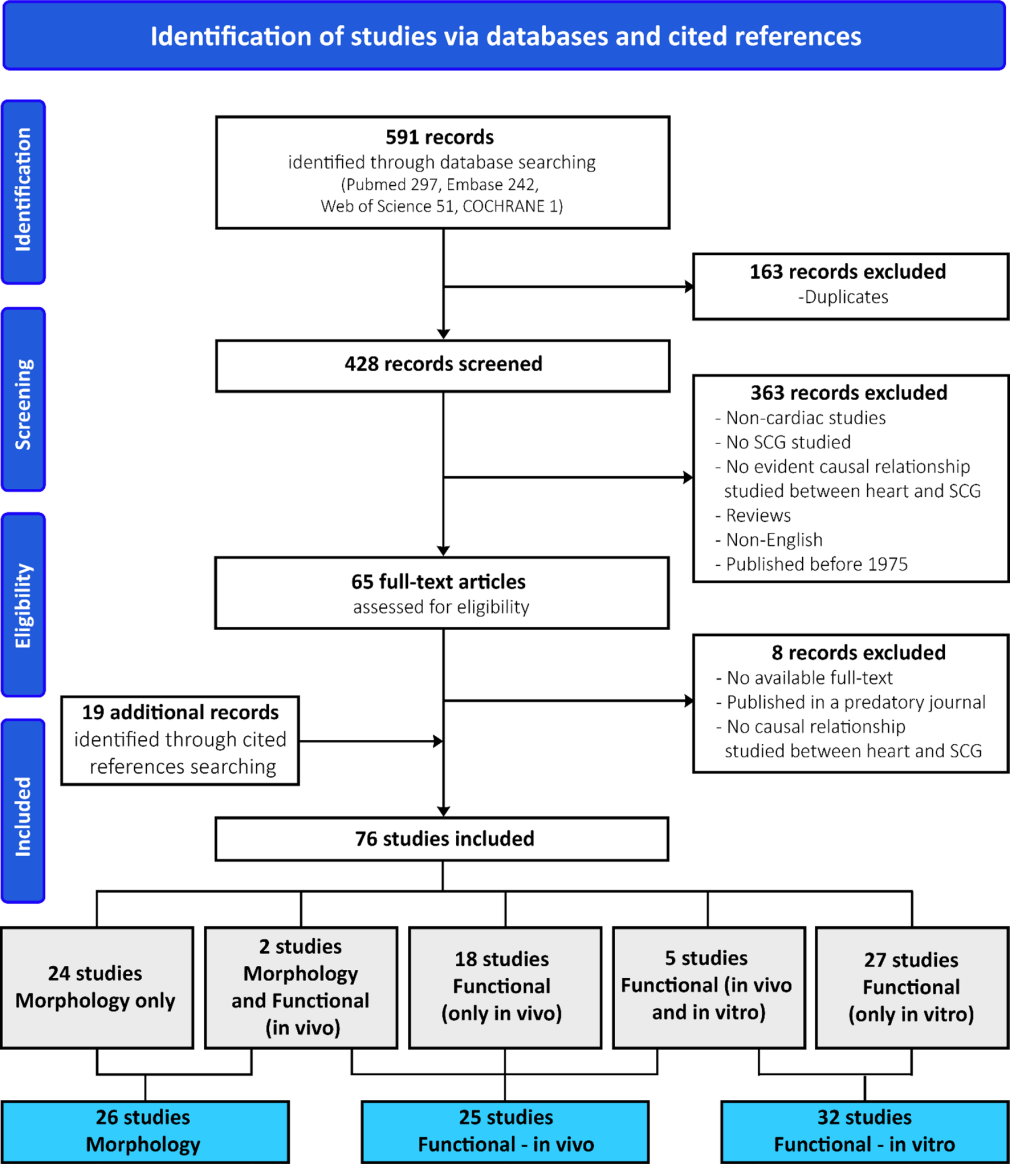
PRISMA flow diagram

A total of 591 records were identified through database searching (Fig. 2). Of these, 163 records were duplicates and the number of studies excluded by abstract screening was 363; most of these did not study both SCG and the heart or lacked an evident study of the causal relationship between the SCG and cardiac tissues. The remaining 65 records were screened based on the full-text articles. Then, 8 records were excluded after full-text assessment and 19 additional records were found through cited references in the remaining articles. A total of 76 studies were included in this systematic review. As some articles fit multiple categories, 26 studies were categorized as morphological, 25 as functional in vivo, and 32 as functional in vitro. Table 1 provides an overview of the studies included in the morphology and functional categories of this systematic review.

**Table 1:** Overview of the studies included in the systematic review

Morphology				Functional							
	Author	Year	Species	In vivo				In vitro			
				No.	Author	Year	Species	No.	Author	Year	Species
1	Kirby et al.	1980	Chick	1	Brandys et al.	1984	Dog	1	Furshpan et al.	1976	Rat
2	Armour and Hopkins	1981	Dog	2	Zhang et al.	2007	Rat	2	Landis	1976	Rat
3	Billman et al.	1982	Nonhuman primate	3	Li et al.**	2010	Rat	3	Chun and Patterson	1977	Rat
4	Hopkins and Armour	1984	Dog	4	Li et al.	2011	Rat	4	King et al.	1978	Rat
5	Shih et al.	1985	Cat	5	Liu et al.*	2013	Rat	5	Schwab and Landis	1981	Rat
6	Janes et al.	1986	Human	6	Kong et al.**	2013	Rat	6	Coughlin et al.	1981	Mouse
7	Wu et al.	1988	Cat	7	Liu et al.**	2014	Rat	7	Coughlin and Kessler	1982	Mouse
8	Pardini et al.	1989	Rat	8	Na et al.	2014	Rat	8	De Ridder and De Potter	1983	Rat
9	Chuang et al.	1992	Nonhuman primate	9	Zhang et al.	2015	Rat	9	Rawdon and Dockray	1983	Mouse
10	Hirakawa et al.	1993	Dog	10	Tu et al.	2016	Rat	10	Kessler et al.	1984	Rat
11	Verberne et al.	1999	Chick/Quail	11	Wu et al.	2016	Rat	11	Uchida and Tomonaga	1985	Mouse
12	Pather et al.	2003	Human	12	Xu et al.	2016	Rat	12	Lahtinen et al.	1986	Rat
13	Chuang et al.	2004	Nonhuman primate	13	Zou et al.	2017	Rat	13	Furshpan et al. (1)	1986	Rat
14	Kawashima and Sasaki	2005	Human	14	Liu et al.	2018	Rat	14	Furshpan et al. (2)	1986	Rat
15	Kawashima	2005	Human	15	Yu et al.*	2018	Rat	15	Potter et al.	1986	Rat
16	Kawashima et al.	2005	Nonhuman primate	16	Ziegler et al.	2018	Mouse	16	Matsumoto et al.	1987	Rat
17	Li et al.	2006	Guinea Pig	17	Cheng et al.**	2018	Rabbit	17	Conforti et al.	1991	Rat
18	Tanaka et al.	2007	Shrew	18	Shi et al.	2019	Rat	18	Rawdon	1991	Mouse
19	Kawashima and Sasaki	2007	Human	19	Prado et al.	2020	Rat	19	Kannan et al.	1994	Rat
20	Kawashima et al.	2008	Nonhuman primate	20	Zhang et al.	2020	Rat	20	Lockhart et al.	1997	Rat
21	Kawashima et al.	2009	Nonhuman primate	21	Zou et al.	2022	Rat	21	Ulupinar et al.	1998	Rat
22	Kawashima and Thorington	2011	Nonhuman primate	22	Ge et al.	2022	Mouse	22	Hasan et al.	2006	Rat
23	Kawashima et al.	2013	Nonhuman primate	23	Cheng et al.**	2023	Rabbit	23	Shcherbakova et al.	2007	Mouse
24	Liu et al.*	2013	Rat	24	Zhang et al.	2023	Rat	24	Li et al.**	2010	Rat
25	Manousiouthakis et al.	2014	Mouse					25	Miwa et al.	2010	Rat
26	Yu et al.*	2018	Rat					26	Takeuchi et al.	2011	Rat
								27	Kong et al.**	2013	Rat
								28	Miwa et al.	2013	Rat
								29	Liu et al.**	2014	Rat
								30	Cheng et al.**	2018	Rabbit
								31	Ge et al.	2020	Mouse
								32	Cheng et al.**	2023	Rabbit

*Morphological and functional in vivo

**Functional in vivo and in vitro

### Quality assessment

**Fig. 3:**
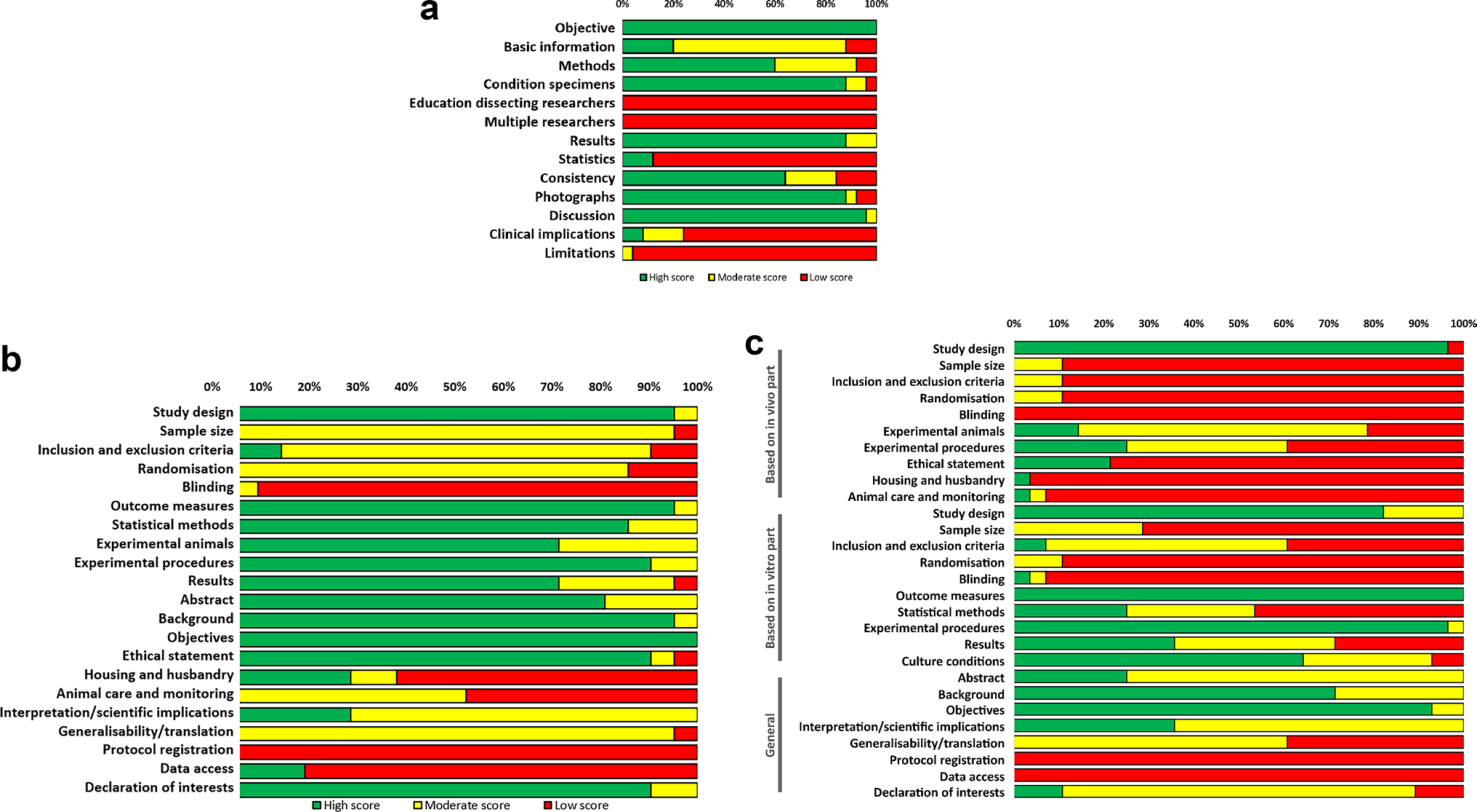
**A** Quality assessment of morphological studies using the QUACS scale. **B** Quality assessment of functional in vivo studies using the ARRIVE guidelines 2.0. **C** Quality assessment of functional in vitro studies using the adjusted ARRIVE guidelines 2.0

Quality scores assessed by QUACS scale ranged from 42% to 65% in morphological studies with a median of 56% (interquartile range 50–61%). In the majority of morphological studies, a thorough description of methods and results was presented, either with or without details on consistency of data with regard to number or percentages of cases in which observations were made (Fig. 3A). In addition, most studies adequately discussed findings in the context of contemporary evidence. Most studies adequately supported their data description with photographs and/or drawings. The relatively low scores could be attributed mainly to deficient data on statistics, education level of dissecting researchers, and the number of observers, which was lacking in all of the included studies. In addition, distinctly indicated study limitations and clinical implications were lacking in most morphological manuscripts.

Applying the ARRIVE guidelines for scoring the functional in vivo studies resulted in quality scores between 43% and 81% with a median of 64% (interquartile range 62–67%). Most detailed the study design, background, objectives, outcome measures, experimental procedures, results, and declaration of interests (Fig. 3B). In contrast to the morphological studies, functional in vivo studies scored high on statistical methods. Similar to the morphological studies, study limitations and clinical implications were lacking in the majority of the functional in vivo manuscripts, affecting the scientific implications and translation items. Furthermore, blinding, protocol registration, and data access were almost completely lacking. Reporting sample size and applying randomization were insufficiently present in the studies. Although ethical statements were present in the majority of the studies, exact details on housing and husbandry conditions and animal care and monitoring were missing.

The range between quality scores of functional in vitro studies assessed by the adjusted ARRIVE guidelines was the greatest (29–68%) with a median of 41% (interquartile range 36–50%). Like the functional in vivo studies, the functional in vitro studies scored high on study design, outcome measures, experimental procedures, background, and objectives (Fig. 3C). Moreover, culture conditions were fairly consistently reported. The low overall scores were mainly owing to the absence of information on the animals that were used to acquire the experimental cells or tissues. The number of cells or tissues used was often lacking or not clearly reported. Some papers were thorough in describing the statistical methods, whereas others lacked information on the software in which the statistical analysis was performed or an explanation of reference to the calculations in case no software was used. Items that were nearly completely lacking in the functional in vivo studies were also insufficient in the functional in vitro studies, such as randomization, blinding, generalizability/translation, protocol registration, and data access. Remarkably, funding was reported in almost all functional in vitro manuscripts, but a declaration of interest was often absent, leading to a moderate score on this item. A detailed quality assessment is presented in the Supplementary Materials (Appendix C).

### Morphological evidence of SCG-cardiac innervation per species

A total of 5 studies were performed in human and 21 in other species (Table 2). In many of the included studies, the main aim was to describe the cardiac autonomic nervous system in general, and they were therefore not focused on the superior cervical ganglion in particular (Supplementary Materials, Appendix D).

**Table 2:** Summary of the key findings in morphological papers

		Morphological studies									
Author	Year	Pre/postnatal	Type of study	N*	Health status	Sex (M/F)	Relevance sex	Both sides SCG included	Relevance sidedness	Direct involvement of SCG in cardiac innervation found	Indirect involvement of SCG in cardiac innervation found
Human											
Janes et al.	1986	Postnatal	Macromorphology	23	Healthy	15/8	NR	Yes	No	No	No
Pather et al.	2003	Prenatal	Macromorphology	8	Healthy	4/4	NR	Yes	No	Yes, via superior cardiac nerve	–
		Postnatal		21		11/10					
Kawashima and Sasaki	2005	Postnatal	Macromorphology	2	Retroesophageal right subclavian artery (cause of death: case 1, gastric cancer; case 2, duodenal cancer)	0/2	–	Yes	Yes, left > right	Sometimes, via superior cardiac nerve	Sometimes, via sympathetic trunk
Kawashima	2005	Postnatal	Macromorphology	18	Clearly abnormal hearts or surrounding vessels excluded	NR/NR	–	Yes	Yes, left > right	Sometimes, via superior cardiac nerve	Sometimes, via sympathetic trunk
Kawashima and Sasaki	2007	Postnatal	Macromorphology	1	Anomalous left vertebral artery; normal great arterial branching pattern of the aortic arch	NR/NR	–	Yes	No	Yes, via superior cardiac nerve	–
				26				Yes	Yes, left > right	Yes, via superior cardiac nerve	
Nonhuman primate (specification see text)											
Billman et al.	1982	Postnatal	Macromorphology	10	Healthy	10/0	–	Yes	No	No	No
Chuang et al.	1992	Postnatal	Retrograde labeling	15	Healthy	Either sex, number NR	NR	Yes	Yes, depending on injected location	Yes, limited number of traced cells	–
Chuang et al.	2004	Postnatal	Retrograde labeling	16	Healthy	Either sex, number NR	NR	Yes	Yes, depending on injected location	Yes, limited number of traced cells	–
Kawashima et al.	2005	Postnatal	Macromorphology	11	Healthy	7/4	No	Yes	No	No	Yes, via sympathetic trunk
Kawashima et al.	2008	Postnatal	Macromorphology	10	Healthy	8/2	No	Yes	Yes, left > right	Sometimes, via superior cardiac nerve	Sometimes, via sympathetic trunk
Kawashima et al.	2009	Postnatal	Macromorphology	12	Clearly abnormal hearts, surrounding great vessels or a heart position associated with a condition excluded	4/7, 1 NR	No	Yes	Yes, left > right	No	Yes, via sympathetic trunk
Kawashima and Thorington	2011	Postnatal	Macromorphology	7	Clearly abnormal heart, surrounding great vessels or a heart position associated with a condition excluded	3/4	NR	Yes	NR	No	Yes, via sympathetic trunk
Kawashima et al.	2013	Postnatal	Macromorphology	3	Healthy	NR/NR	–	Yes	No	No	Yes, via sympathetic trunk
Dog											
Armour and Hopkins	1981	Postnatal	Retrograde labeling	38	Healthy	Either sex, number NR	NR	Yes	Yes, depending on injected location	Yes, limited number of traced cells	–
Hopkins and Armour	1984	Postnatal	Retrograde labeling	27	Healthy	Either sex, number NR	NR	Yes	Yes, depending on injected location	Yes, limited number of traced cells	–
Hirakawa et al.	1993	Postnatal	Retrograde labeling	23	Healthy	Either sex, number NR	NR	Yes	Yes, depending on injected location	Yes, limited number of traced cells	–
Cat					Healthy						
Shih et al.	1985	Postnatal	Retrograde labeling	15	Healthy	Either sex, number NR	NR	Yes	Yes, depending on injected location	Yes, limited number of traced cells	–
Wu et al.	1988	Postnatal	Retrograde labeling	28	Healthy	Either sex, number NR	NR	Yes	Yes, depending on injected location	Yes, limited number of traced cells	–
Guinea pig											
Li et al.	2006	Postnatal	Anterograde labeling	NR	Chemical sympathectomy with 6-OHDA	NR/0	–	No	–	Yes, limited number of traced cells	–
Rat											
Pardini et al.	1989	Postnatal	Retrograde labeling	69	Healthy	NR/NR	–	Yes	No	Yes, limited number of traced cells	–
Liu et al.	2013	Postnatal	Retrograde labeling	24	MI by LAD occlusion	NR/NR	–	No, only left SCG	–	Yes, limited number of traced cells	–
Yu et al.	2018	Postnatal	Retrograde labeling	24	MI by LAD occlusion	24/0	–	Yes	No	Yes, limited number of traced cells	–
Mouse											
Manousiouthakis et al.	2014	Prenatal	Histomorphology	Unclear	Healthy	NR/NR	–	Yes	NR	No	Yes, intermixed with projections from stellate ganglia
Shrew											
Tanaka et al.	2007	Postnatal	Macromorphology	10	Healthy	5/5	NR	Yes	No	No	Yes
Chick											
Kirby et al.	1980	Prenatal	Histomorphology	NR chick	Healthy	NR/NR	–	Yes	No	No	No
Verberne et al.	1999	Prenatal	Histomorphology	3 chick, 21 quail-chick chimeras	Healthy	NR/NR	–	Yes	NR	No	Yes, via carotid nerve

Only *n*-numbers related to SCG were included in the table.* NR* Not reported, when health state is not specifically reported, it was considered as healthy

#### Human (*n* = 5)

The cardiac nerves are commonly described as nerves connecting to the heart either “with direct connections or connections via the cardiac plexus” [2, 9, 16, 25]. The superior cardiac nerve (SCN) was observed in most studies [9, 25–27]. When observed, the SCN arose directly from the SCG (53–100% of cases) or the sympathetic trunk between the SCG and middle cervical ganglia (MCG). The left and right SCG were investigated in all included studies, but there is no consensus about the relevance of sidedness. Some subjects showed no relevance of sidedness [26, 27], while in others a larger contribution of the left than the right SCG was seen [9, 25, 27]. In one study, no cardiopulmonary nerves were found to originate from either the SCG or the sympathetic trunk between the SCG and MCG [28]. The studies included subjects from both sexes [26] or only females [25], or did not report the sex [9, 27]. Only one study also investigated prenatal subjects (8 out of 29 subjects) but did not specifically report separate results on the contribution of the SCG to cardiac innervation in pre- and postnatal/adult subjects [26].

#### Nonhuman primate (*n* = 8)

The relation between the SCG and cardiac innervation has been studied in various species of nonhuman primates (arranged by primate evolutionary phylogeny): Lorisiformes (lorises and galagos) [29], tarsiers [30], New World Monkeys [31], Old World Monkeys (e.g., rhesus, macaque, and Taiwan monkey) [32–34], gibbons [35], and unspecified [36].

**Fig. 4:**
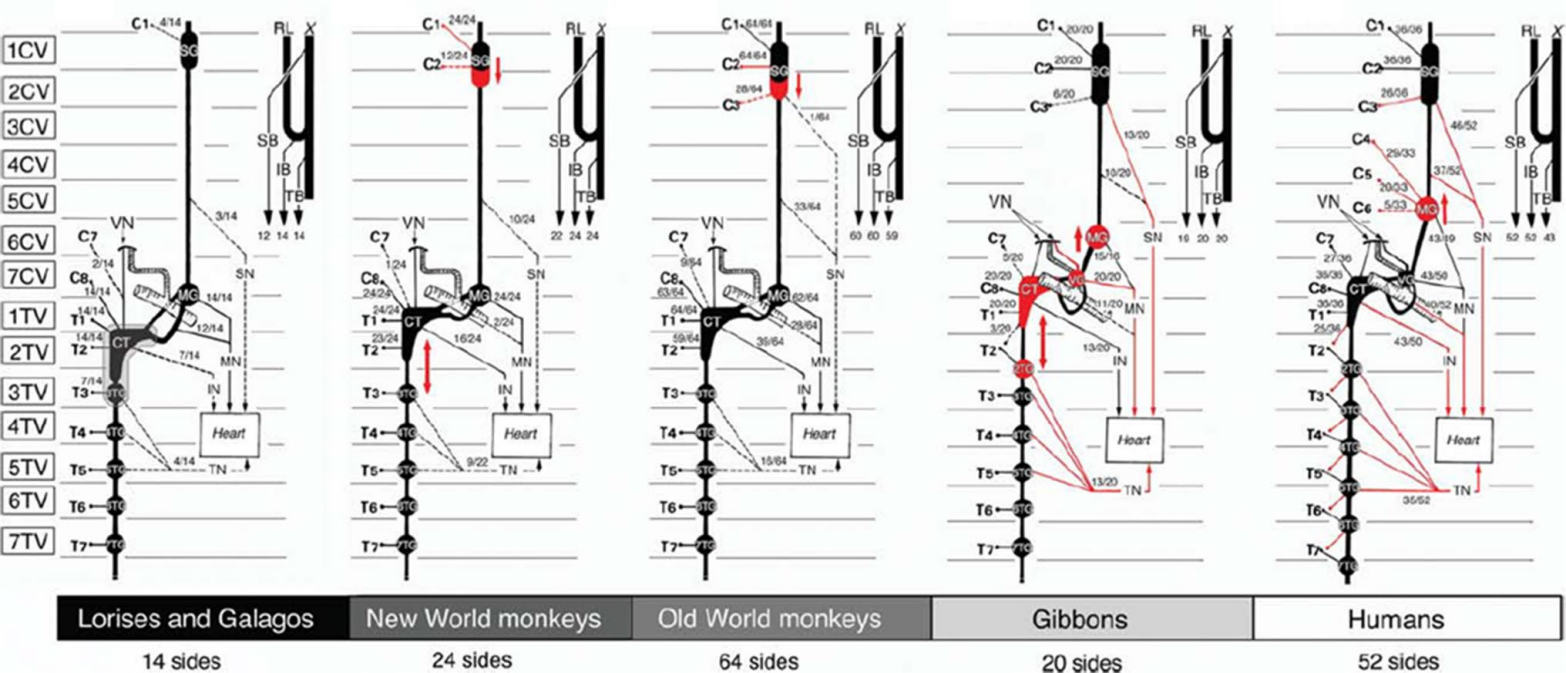
Evolutionary overview of cardiac sympathetic innervation. This figure is derived from “Comparative morphological configuration of the cardiac nervous system in lorises and galagos (Infraorder Lorisoformes, Strepsirrhini, Primates) with evolutionary perspective,” by Thorington and Kawashima. *The Anatomical Record: Advances in Integrative Anatomy and Evolutionary Biology*, 2011 Mar;294(3):412–26. Copyright 2011 by Copyright Clearance Center

In the Lorisiformes, tarsiers, New World Monkeys, and Old World Monkeys, the SCN was observed, but never originated directly from the SCG. Instead, the SCN was found to originate indirectly from the sympathetic trunk between the SCG and MCG [29–31, 33]. Contrastingly, one study performed in Old World Monkeys did not find a visual direct nor indirect connection between the SCG and the heart [32]. However, retrograde labeling with horseradish peroxidase (HRP) supported nerve connection between the SCG and the heart, also in Old World Monkeys [34, 36]. In gibbons, a nonhuman primate that is evolutionarily closed to the human, the SCN was observed to originate from the SCG in 65% and from the sympathetic trunk below the SCG in 50% of cases [35]. Within primates, higher levels in the evolutionary hierarchy seemed to relate to increased prevalence of the superior cardiac nerve and increased observation of a direct connection from the superior cardiac nerve toward the heart (Fig. 4).

Most studies included subjects from both sexes [29, 31, 33, 35], although the exact number per sex was sometimes not reported [36]. One study included only males, and one study did not report on the sex [30, 32]. In case results were specified per sex, no evident differences existed [31, 33, 35]. Both sides of the SCG were included in all studies, but the relevance of sidedness remains dubious, as some reported no differences [30, 32, 33], while others found a dominance of the left SCG over the right SCG, or reported that relevance of sidedness depended on the injected location of HRP [31, 34–36]. No prenatal studies in the nonhuman primates investigating the connections between SCG and the heart were found.

#### Dogs (*n* = 3)

All studies performed in postnatal dogs used retrograde labeling to trace the connectivity from the heart to the SCG and found an involvement of the SCG in cardiac innervation, although the contribution was minimal [37–39]. Only very few neurons could be traced back in the SCG after HRP injection in the heart or cardiac nerves, and in more than half of the SCG, no retrogradely labeled neurons could be traced back at all. The dogs were from either sex, but the number and the results were not reported separately. Both sides of the SCG were included in all studies, but the results were different depending on the location of the HRP injection. Upon HRP injection into the cardiac nerves, only sporadically labeled neurons were found in the caudal pole of the ipsilateral SCG [39]. No labeled neurons were observed on the contralateral side. After injection in the heart, only a small number of labeled cells could be traced back in the bilateral SCG [37, 38].

#### Cats (*n* = 2)

In both morphological studies performed in postnatal cats, retrograde labeling was used to study the relation between the heart and SCG [40, 41]. Data in the cat show similar results as in dogs, with no or only very few cells that could be traced back into the SCG after injection of HRP into the heart, depending on the injected location. Likewise, both SCG sides were included and its relevance depended on the site of HRP injection. Subjects were from either sex, but the exact number and the results were again not specified for each sex.

#### Guinea pigs (*n* = 1)

Only one study focused on the morphology of SCG in cardiac innervation in postnatal guinea pigs using anterograde labeling [42]. The tracer biotinylated dextran amine (BDA) was injected into the SCG, and a distribution of anterogradely traced sympathetic axons and varicosities was observed in the heart. No conclusions can be drawn regarding sex and SCG sidedness, as only males were included in the study and the SCG side was not reported.

#### Rats (*n* = 3)

All papers investigated the morphological relevance of the SCG in cardiac innervation in postnatal rats using retrograde labeling [43–45]. Again, a small morphological contribution of the SCG in cardiac innervation seems to be present. Only a small minority of labeled cells were found bilaterally in the SCG after injection of the heart (3% left ventricle, 1% right ventricle). Interestingly, HRP injected into the cardiac apex several days after MI revealed more HRP staining in the SCG from MI rats compared with control or sham [43, 45]. One study was performed in male rats [45], while the sex was not reported in the other studies [43, 44]. In the two studies in which both sides of the SCG were included, no evident differences were found between the sides [44, 45].

#### Mice (*n* = 1)

Through murine whole-mount visualization of sympathetic nerve morphology using tyrosine hydroxylase (TH) staining, it was observed that a portion of nerves arising from the SCG project toward the heart [46]. These nerves intermix on the ventral side of the heart with the ventral projections from the stellate ganglia. Surprisingly, the SCG nerves did not project to the dorsal side of the heart. Both sides of the SCG were included, but the results were not specified per side. The sex was also not reported. In contrast to the previously described species, only prenatal mice were studied.

#### Shrew (*n* = 1)

Macromorphological connections between SCG and heart were present in the postnatal shrew [47]. The nerve originating from both the left and right SCG descended to reach the aortic arch and formed nerve plexuses supplying nerves to the ventral wall of the ventricle. An equal number of male and female shrews were studied, but the results were not specified per sex.

#### Chick (*n* = 2)

Histomorphological techniques (catecholamine histofluorescence and silver preparations) were used to study the developing sympathetic innervation in chick embryos [48]. All nerve branches from the SCG were directed cranially, and no fluorescent postganglionic fibers could be traced from the SCG to the heart. In contrast, in another study, no direct connection between the SCG and the heart was found, but the SCG seemed to innervate the heart indirectly via the carotid nerve, which joins the nodose ganglion of the vagal nerve whose branches enter the arterial and venous pole of the heart [49]. Both sides of the SCG were included in both studies, but the results were not separately reported. Additionally, the sex was not reported.

#### Summary

- In all species, morphological evidence that the SCG innervates the heart either directly or indirectly was found, but its contribution is likely limited as only very few neurons could be retrogradely traced.
- SCG sidedness may be relevant, and if so, often the left SCG is macromorphologically considered to contribute more than the right SCG to cardiac innervation. Of note, findings depended on the site of injection in the heart in retrograde labeling studies.
- There is not enough morphological information about sex differences.
- Prenatal studies were found to be underrepresented in morphological studies.

### Functional in vivo evidence of SCG-cardiac innervation per species

Studies in the functional in vivo category were predominantly performed in rats (Table 3). All studies only included the postnatal/adult stage. The functional relationship between SCG and the heart in vivo was often part of the primary aim (Supplementary Materials, Appendix E).

**Table 3:** Summary of the key findings in functional in vivo papers

Functional—in vivo studies												
Author	Year	Pre/postnatal	N*	Strain/breed	Condition	Sex (M/F)	Relevance sex	Both sides SCG included	Relevance sidedness	Substances tested/outcome measures	Any direct involvement of SCG in cardiac innervation found	Any indirect involvement of SCG in cardiac innervation found
Dog												
Brandys et al.	1984	Postnatal	4	Mongrel	Hexamethonium administration	Either sex, number NR	NR	Yes	No	Heart rate, atrial contraction force, intramyocardial pressure, ECG	No	–
Rabbit												
Cheng et al.	2018	Postnatal	10	NR	Isoproterenol-induced MI	Either sex, number NR	NR	NR	–	Fluvastatin	–	Yes
Cheng et al.	2023	Postnatal	18	NR	Isoproterenol-induced MI	18/0	–	NR	–	P2Y_12_, TH, ECG, echo	–	Yes
Rat												
Zhang et al.	2007	Postnatal	Unclear	Sprague–Dawley	Isoproterenol-induced MI	Unclear/0	–	NR	–	A-317491, P2X_3_, behavior	–	Yes
Li et al.	2010	Postnatal	Unclear	Sprague–Dawley	Isoproterenol-induced MI	Either sex, number NR	NR	NR	–	A-317491, P2X_2_, P2X_3_, TH, blood pressure, heart rate, respiration	–	Yes
Li et al.	2011	Postnatal	Unclear	Sprague–Dawley	Isoproterenol-induced MI	Either sex, number NR	NR	NR	–	Oximatrine, NE, P2X_3_, blood pressure, heart rate	–	Yes
Liu et al.	2013	Postnatal	64	Sprague–Dawley	LAD occlusion-induced MI	NR	–	NR	–	OxATP, TNFα, IL-6, CK-MB, CK, LDH, cardiac troponin I, glutamine synthetase, ERK, TH, substance P, neuronal nuclei, ECG (Q wave), blood pressure, heart rate	–	Yes
Kong et al.	2013	Postnatal	56	Sprague–Dawley	LAD occlusion-induced MI	56/0	–	NR	–	BBG, P2X_7_, _(p-)_ERK1/2	–	Yes
Liu et al.	2014	Postnatal	30	Sprague–Dawley	Isoproterenol-induced MI	Either sex, number NR	NR	NR	–	Puerarin, P2X_3_, TH, blood pressure, heart rate	–	Yes
Na et al.	2014	Postnatal	30	Sprague–Dawley	Monocrotaline-induced pulmonary hypertension with left SCG block	30/0	–	No, only left	–	Nitrite, superoxide dismutase, RV systolic pressure, RV/LV mass ratio, heart rate variability	–	Yes
Zhang et al.	2015	Postnatal	25	Sprague–Dawley	LAD occlusion-induced MI	25/0	–	NR	–	Baicalin, P2X_3_, CK-MB, cardiac troponin T, epinephrine, ATP, cardiac hypertrophy, Q wave (ECG), blood pressure, heart rate,	–	Yes
Tu et al.	2016	Postnatal	56	Sprague–Dawley	LAD occlusion-induced MI	Either sex, number NR	NR	NR	–	NONRATT021972 siRNA, baicalin, P2X_7_, GAP43, TH, ECG (Q wave), blood pressure, heart rate	–	Yes
Wu et al.	2016	Postnatal	24	Sprague–Dawley	Diabetes mellitus	24/0	–	NR	–	Uc.48 + siRNA, P2X_2_, P2X_3_, P2X_5_, P2X_7_, _(p-)_ERK1/2	–	Yes
Xu et al.	2016	Postnatal	24	Sprague–Dawley	Diabetes mellitus	24/0	–	NR	–	NONRATT021972 siRNA, TNFα, insulin receptor substrate 1, neuronal nuclei, heart rate variability	–	Yes
Zou et al.	2017	Postnatal	40	Sprague–Dawley	LAD occlusion-induced MI	NR	–	NR	–	P2Y_12_ shRNA, P2Y_12_, GFAP, TNFα, _(p-)_P38 mitogen-activated protein kinase, myocardial fiber structure, blood pressure, heart rate	–	Yes
Liu et al.	2018	Postnatal	40	Sprague–Dawley	Β-aminopropionitrile-induced aortic dissection with SCG removal	40/0	–	Yes, bilateral	–*	Matrix metalloproteinase-9, aortic wall thickening, heart rate, aortic dissection incidence	–	Yes
Yu et al.*	2018	Postnatal	24	Sprague–Dawley	LAD occlusion-induced MI	24/0	–	NR	–	Oxytonergic receptor, TH	–	Yes
Shi et al.	2019	Postnatal	Unclear	Sprague–Dawley	LAD occlusion-induced MI	Unclear/0	–	NR	–	TH, GAD65/67, GABA_A_Rβ2, P2X_7_, intracellular Ca^2+^, NE, renal sympathetic nerve activity	–	Yes
Prado et al.	2020	Postnatal	Unclear	Wistar	MI with reperfusion with SCG removal (Langendorff-setting)	Unclear/0	–	Yes, bilateral	–*	Melatonin receptor, SERCA2A, K-ATP channels, connexin-43, TNFα, nitrotyrosine, TGFβ, vimentin, ECG (ventricular arrhythmias, QRS, QT, QTc, PR), epicardial action potential, early after depolarizations, heart rate	–	Yes
Zhang et al.	2020	Postnatal	104	Sprague–Dawley	LAD occlusion-induced MI	104/0	–	NR	–	SCG10 (marker for axonal regeneration), TH	–	Yes
Zou	2022	Postnatal	72	Sprague–Dawley	LAD occlusion-induced MI	72/0	–	NR	–	Uc.48 + lncRNA/shRNA, clopidogrel, heart rate, blood pressure	–	Yes
Zhang	2023	Postnatal	40	Sprague–Dawley	LAD occlusion-induced MI	40/0	–	NR	–	P2X_7_, GS, ECG, sympathetic nerve discharge (SND), blood pressure	–	Yes
Mouse												
Ziegler et al.	2018	Postnatal	22	C57BL6/N	LAD occlusion-induced MI with SCG removal	22/0	–	Yes, bilateral	–*	Sirius Red, high-sensitivity cardiac troponin T, pro-NGF, TH, CD68, Cx3cr1, TNFα, cardiac hypertrophy, cardiomyocyte hypertrophy, cardiac function	–	Yes
Ge et al.	2022	Postnatal	20	C57BL/6 J	LAD occlusion-induced MI	20/0	–	Yes	No	NGF, BDNF, GAP43, TrK, TH, CHAT, neuron size, beta-III-tubulin	–	Yes

*HRV* heart rate variability; *ISH* in situ hybridization, *NR* not reported, *SCGx* superior cervical ganglionectomy, *A-317491* selective P2X2/3 receptor antagonist, oxATP: P2X7 receptor antagonist, *BBG* Brilliant Blue G: P2X7 receptor antagonist, puerarin: major active ingredient extracted from the traditional Chinese plant medicine Ge-gen, Baicalin: compound purified from the dry roots of *Scutellaria baicalensis*

*Owing to bilateral SCGx, the relevance of sidedness could not be studied

#### Dogs (*n* = 1)

The direct role of the SCG in cardiac innervation was investigated by measuring cardiac responses to stimulation of sympathetic ganglia in dogs from either sex (not further specified) [50]. When the SCG were stimulated bilaterally in a variety of regions, none of the stimulations produced any detectable cardiac responses. These data indicate that, although morphologically a small amount of nerves can be traced between heart and SCG, functionally this is insufficient to achieve significant cardiac stimulation.

#### Rabbits (*n* = 2)

After the induction of MI in rabbits, ion channel characteristics of SCG neurons were studied. It was found that various channel proteins were increased in the SCG neurons after MI, indicating that a functional link between the SCG and heart exists in vivo [51]. The rabbits were reported to be from either sex, but these results were not reported separately. Additionally, which SCG side was included in the experiments was not reported. In another study of male rabbits, MI led to alterations in the activation and inactivation characteristics of the sodium channels accompanied by increase expression of P2Y12, a purinergic receptor, in SCGs [52]. Of note, these changes in the SCG post-MI do not necessarily show a direct connection, but could be indirect owing to alterations in SCG function through reflex pathways.

#### Rats (*n* = 21)

A vast amount of functional in vivo evidence for the indirect contribution of the SCG to cardiac innervation can be attributed to the multitude of studies performed in rats. Different functional methods were used, such as the measurement of blood pressure and heart rate, combined with (immuno)histochemistry, immunofluorescence, Western Blot, (q)RT-PCR, enzyme-linked immunosorbent assay (ELISA), and in situ hybridization. All studies found a functional in vivo connection between the SCG and the heart. This link could be observed in various pathological conditions, including MI (induced by isoproterenol or LAD occlusion), diabetes mellitus, aortic dissection, and pulmonary hypertension (Table 3). Many changes in the SCG after MI were related to purinergic signaling [43, 53–63]. The studies included male rats or rats from either sex, but in the latter case the exact number per sex was not reported. Studies only performed in female rats did not exist. In nearly all studies, the side of the studied SCG was not reported.

#### Mice (*n* = 2)

Both studies were performed in male adult C57BL6 mice with MI induced by LAD occlusion [64, 65]. Interestingly, removal of the SCG led to almost entire loss of myocardial sympathetic innervation in the left anterior wall (devoid of immunoreactivity) and the left and right SCG comparably contributed to the innervation of the left anterior wall [64]. Neuronal remodeling toward an increased adrenergic phenotype was observed in the SCG after MI, and these changes did not seem to differ between the left and right SCG [65]. When bilaterally removing the SCG during MI, positive effects were seen regarding cardiac function, inflammation, and hyperinnervation [64].

#### Summary

- Functional in vivo evidence indicates that the SCG indirectly contributes to cardiac innervation and could be attributed to involvement in sympathetic overdrive reactions in response to cardiac diseases. Most data are derived from studies in disease models. A study performed in dogs failed to demonstrate a direct contribution.
- Information on the relevance of SCG sidedness is largely unavailable.
- The female sex is highly underrepresented in functional in vivo studies, and no information on the relevance of sex exists.
- All studies were performed in the postnatal stage. No functional in vivo studies were found in the prenatal stage.

### Functional in vitro evidence of SCG-cardiac innervation per species

Similar to the functional in vivo category, functional in vitro studies were predominantly performed in rats (Table 4). The assessment of a functional relationship between SCG and the heart in vitro was often part of the primary aim (Supplementary Materials, Appendix F).

**Table 4:** Summary of the key findings in functional in vitro papers

Functional—in vitro studies															
Author	Year	Prenatal/postnatal/mixed	N*	Strain/breed	Condition	Sex (M/F)	Relevance sex	Both sides SCG included	Relevance sidedness	Evaluation methods	Substances of interest	Experimental setting		Any direct involvement of SCG in cardiac innervation found	Any indirect involvement of SCG in cardiac innervation found
Rabbit															
Cheng et al.	2018	Postnatal	70	NR	Isoproterenol-induced MI	Either sex, number NR	NR	NR	–	Electrophysiological recordings	Fluvastatin	Damage to the myocardium, isolated SCG neurons studied	–		Yes
Cheng et al.	2023	Postnatal	NR	NR	Isoproterenol-induced MI	NR	–	NR	–	Electrophysiological recordings	P2Y_12_	Damage to the myocardium, isolated SCG neurons studied	–		Yes
Rat															
Furshpan et al.	1976	Postnatal	180	NR	Healthy	NR	–	NR	–	Electrophysiological recordings	Atropine, propranolol, hexamethonium	Co-culture	–		Yes*
Landis	1976	Postnatal	NR	NR	Healthy	NR	–	NR	–	Electron microscopy	–	Co-culture	–		Yes*
Chun and Patterson	1977	Postnatal	NR	NR	Healthy	NR	–	NR	–	Phase-contrast microscopy, isotopic assay	NGF	Co-culture	–		Yes
King et al.	1978	Postnatal	NR	Wistar	Healthy	NR	–	NR	–	Optical recordings	Tyramine, norepinephrine	Co-culture	–		Yes
Schwab et al.	1981	Postnatal	Unclear	NR	Healthy	NR	–	NR	–	Electron microscopy, fluorescence microscopy	Lectins, toxins, HCM	Culture of SCG neurons with HCM	–		Yes*
De Ridder and De Potter	1983	Prenatal	NR	Sprague–Dawley	Healthy	NR	–	Yes	NR	Electron/phase-contrast/fluorescence microscopy	–	Co-culture	–		Yes*
Kessler et al.	1984	Postnatal	8	Sprague–Dawley	Healthy	NR	–	NR	–	Phase-contrast microscopy, radioimmunoassay, HPLC	Substance P	Co-culture	–		No
Lahtinen et al.	1986	Mixed	NR	Sprague–Dawley	Healthy	Either sex, number NR	NR	NR	–	Phase-contrast microscopy	NGF	Co-culture	–		Yes
Furshpan et al. (1)	1986	Postnatal	NR	Albino	Healthy	NR	–	NR	–	Electrophysiological recordings, electron microscopy	Acetylcholine, adenosine, alprenolol, atenolol, atropine, hexamethonium, methylxanthines, norepinephrine, phentolamine, propanolol, reserpine, sotalol	Co-culture	–		Yes*
Furshpan et al. (2)	1986	Postnatal	NR	Albino	Healthy	NR	–	NR	–	Electrophysiological recordings	Acetylcholine, adenosine, atenolol, atropine, methylxanthines, norepinephrine, phentolamine,	Co-culture	–		Yes*
Potter et al.	1986	Postnatal	NR	Albino	Healthy	NR	–	NR	–	Electrophysiological recordings, electron microscopy	Acetylcholine, adenosine, alprenolol, atenolol, atropine, hexamethonium, methylxanthines, norepinephrine, phentolamine, propanolol, reserpine, sotalol, HCM, HCM factor	Co-culture	–		Yes*
Matsumoto et al.	1987	Postnatal	NR	NR	Healthy	NR	–	NR	–	Electrophysiological recordings	Acetylcholine, adenosine, atenolol, atropine, hexamethonium, serotonin–creatinine sulfate, gramine, methylxanthines, methysergide, phentolamine, Peptide YY, reserpine, somatostatin, substance P, neurotensin, VIP	Co-culture	–		Yes
Conforti et al.	1991	Postnatal	Unclear	Sprague–Dawley	Healthy	NR	–	NR	–	Electrophysiological recordings	Nifedipine	Co-culture	–		Yes
Kannan et al.	1994	Postnatal	NR	Wistar	Healthy	Either sex, number NR	NR	NR	–	Phase-contrast microscopy	NGF	Co-culture	–		Yes
Lockhart et al.	1997	Postnatal	221	Simonson White	Healthy	NR	–	NR	–	Electrophysiological recordings, phase-contrast/fluorescence microscopy, RT-PCR	NGF, norepinephrine, TrkA	Co-culture	–		Yes
Ulupinar et al.	1998	Mixed	10	Sprague–Dawley	Healthy	NR	–	NR	–	Phase-contrast microscopy, DiI-labeling	-	Co-culture	–		Yes
Hasan	2006	Postnatal	Unclear	Sprague–Dawley	LAD occlusion-induced MI	Female	–	NR	–	Phase-contrast microscopy	NGF	Co-Culture	–		Yes
Li et al.	2010	Postnatal	18	Sprague–Dawley	Isoproterenol-induced MI	Either sex, number NR	NR	NR	–	Electrophysiological recordings	ATP, A-317491	Damage to the myocardium, isolated SCG neurons studied	–		Yes
Miwa et al.	2010	Postnatal	Unclear	Wistar	Healthy	NR	–	NR	–	Electron/fluorescence microscopy	NGF, BDNF, GDNF, CNTF, Sema3A	Co-culture	–		Yes
Takeuchi et al.	2011	Postnatal	Unclear	Wistar	Healthy	NR	–	NR	–	Electrophysiological recordings	Propanolol	Co-culture	–		Yes*
Kong et al.	2013	Postnatal	Unclear	Sprague–Dawley	LAD occlusion-induced MI	Male	–	NR	–	Electrophysiological recordings	P2X_7_, BzATP, BBG, GF109203X	Damage to the myocardium, isolated SCG neurons studied	–		Yes
Miwa et al.	2013	Postnatal	91	Wistar	Healthy	NR	–	NR	–	Electrophysiological recordings, electron/fluorescence microscopy	NGF, GDNF, synapsin-1	Co-culture	–		Yes
Liu et al.	2014	Postnatal	NR	Sprague–Dawley	Isoproterenol-induced MI	Either sex, number NR	NR	NR	–	Electrophysiological recordings	Puerarin, A-317491	Damage to the myocardium, isolated SCG neurons studied	–		Yes
Mouse															
Coughlin et al.	1981	Prenatal	Unclear	CD-1	Healthy	NR	–	NR	–	Phase-contrast microscopy, biochemistry	HCM factor, TH, CHAT, NGF	Culture of whole SCG with HCM factor	–		Yes*
Coughlin and Kessler	1982	Prenatal	NR	CD-1	Healthy	NR	–	NR	–	Chromatography, electrophoresis	NGF, HCM factor	Culture of whole SCG with HCM factor	–		Yes
Rawdon and Dockray	1983	Postnatal	Unclear	Piebald-lethal	Healthy	NR	–	NR	–	Phase-contrast microscopy	–	Co-culture	–		Yes
Uchida and Tomonaga	1985	Mixed	NR	C57BL/6	Healthy	Male	NR	NR	–	Phase-contrast microscopy	NGF, HCM	Culture of SCG neurons with HCM	–		Yes
Rawdon	1991	Mixed	24	Swiss Webster	Healthy	NR	–	NR	–	Phase-contrast microscopy	NGF	Co-culture	–		Yes
Shcherbakova et al.	2007	Postnatal	NR	NR	Healthy	NR	–	NR	–	Fluorescence microscopy, optical recordings	Cadherins, catenins, SAP97, AKAP79, b1Ars, caveolin-3, b2Ars, norepinephrine, epinephrine	Co-culture	–		Yes
Ge et al.	2020	Mixed	81	C57BL/6 J	Healthy	Either sex, number NR	NR	NR	NR	Fluorescence microscopy, PCR, western blot	β3-Tubulin, NGF	Co-culture	–		Yes

*Con A* concanavalin A, *WGA* wheat germ agglutinin, *SBA* soybean agglutinin, *HA* hemagglutinin, *HLPC* high-performance liquid chromatography, *8-PT* 8-phenyltheophylline, *VIP* vasoactive intestinal polypeptide, *BBG* Brilliant Blue G: P2X7 receptor antagonist, GF109203X: protein kinase C inhibitor, A-317491: selective P2X2/3 receptor antagonist, *HCM* heart-conditioned medium

*No control group was included to verify the results

#### Rabbit (*n* = 2)

After studying the functional characteristics of ion channels in SCG of postnatal MI rabbits in vivo, the electrical activity of SCG neurons was also studied in vitro after MI [51, 52]. The mean amplitude of action potentials of the neurons increased and action potential duration (APD90) was shorter after MI (Supplementary Materials, Appendix F). As mentioned above, the rabbits could be from either sex (not further specified) and SCG side was not reported.

#### Rat (*n* = 23)

Regarding experiments performed in rats, co-cultures of myocardium and neurons were most common, followed by measurements in isolated SCG neurons after damage to the myocardium or culture of SCG neurons using heart conditioned medium (Table 4). A great diversity in co-culture methods were reported, including:

1. Single SCG neurons or multiple dissociated SCG neurons on top of cardiomyocytes
2. Small SCG tissue clumps or whole SCG explants attached to or cultured in close proximity to cardiomyocytes/myocardium
3. Mass culture of dissociated neurons and cardiomyocytes

Of importance, it should be noted that co-culture experiments are limited to investigating the influence of cell types on each other. This means that only an indirect link between the SCG and the heart can be investigated through co-culture experiments, but a direct link can not be unequivocally determined.

Off all included studies, only one study did not indicate a contribution of the SCG to cardiac innervation [66]. In that study, dissociated SCG neurons co-cultured with monolayers heart cells had no effect on neuropeptide content, while co-cultures with the pineal and salivary gland resulted in a striking increase.

In contrast, all other studies on a functional in vitro level supported a role for the SCG in cardiac innervation. SCG neurons co-cultured with cardiomyocytes resulted in the survival of SCG neurons, the development of functional synaptic contacts and in addition, cardiomyocytes reacted by evoked responses and increasing beat rate upon stimulation of the SCG neurons [67–74]. Individual neurons within the SCG could have differential effects on the cardiomyocytes (inhibitory, excitatory, and dual function) by secreting different neurotransmitters [75–77]. The functional in vivo role of purinergic signaling in the interplay between SCG and the heart was also confirmed in vitro, where many of the SCG neurons could evoke hyperpolarizations of cardiomyocytes, which were attenuated by adenosine-receptor blockers and adenosine deaminase, an enzyme that hydrolyzes adenosine to pharmacologically inactive inosine [78]. Similar evidence for a functional relation between SCG and the heart was found in experiments using small SCG tissue clumps and whole SCG explants. Co-culturing heart tissue with SCG caused stimulation and directional orientation of neurite outgrowth of SCG, while this was less evident in co-cultures of myocardium with other types of tissue [79–82]. In three studies, SCG neurons were cultured after damage to the myocardium by induction of MI [55, 57, 58]. These studies revealed that MI led to pathological changes in the electrophysiological properties of SCG neurons, indicating a relation between SCG and the heart. Interestingly, the addition of heart conditioned medium induced remodeling of the SCG neurons in the form of fundamental changes in the phenotype (adrenergic/cholinergic/purinergic) as well as in the secreted neurotransmitter [77, 83]. Controls for heart tissue or cells often comprised tissue or cells from another origin, such as the gut. Of note, close to a third of all functional in vitro studies performed in rats did not use a control group for cardiomyocytes or neurons to verify the results [67, 74–78, 81, 83].

Co-cultures of SCG and hearts that were both derived from prenatal rats were used in only one study [81]. Two studies used prenatal SCG, while culturing it with postnatal cardiac tissue [79, 82]. All other studies were performed with SCG and hearts from postnatal rats (19 out of 22; Table 4). The macromorphological or functional in vivo category the postnatal stage in the functional in vitro category mainly consisted of neonates instead of adults. Sporadically, it was stated that the neurons or myocytes derived from the male sex or either sex, while the exact numbers were not specified. Potential sex differences were not reported in the results [55, 57, 58, 79, 80], The sidedness of the SCG was not reported in the included studies in rat.

#### Mice (*n* = 7)

All SCG (co-)cultures performed with murine material supported the presence of a neuron–cardiomyocyte relationship. Similar to the experiments performed in rats, directional outgrowth of neurites from whole murine SCG was observed when co-cultured with heart tissue [84, 85]. Additionally, co-culturing cardiomyocytes on a layer of dissociated SCG neurons revealed the formation of functional synapses [86]. Stimulation of neuronal activity resulted in changes in sympathetic receptors in the cardiomyocytes, supporting the role of the SCG to cardiac innervation. Adding heart conditioned medium to dissociated SCG neurons induced neurite survival, including production and elongation [87, 88]. On the other hand, antiserum to a neuronal growth factor isolated from heart conditioned medium specifically blocked neurite extension [89]. These findings support the presence of a substance in heart conditioned medium influencing the SCG.

Two studies included postnatal mice [84, 86], two papers included prenatal mice [87, 89], and three used a combination of pre- and postnatal mice [85, 88, 90]. Of these studies, one was performed in tissue from adult mice ranging from 6 to 30 months; postnatal animals in other studies were from the neonatal stage. Direct comparisons between the pre- and postnatal stage in the SCG to cardiac innervation are scarce, and owing to the heterogeneity in the exact age and culture settings, it is currently impossible to draw conclusions regarding differences in pre- and postnatal stage.

The male sex was only stated in one study [88], while all other studies did not report the sex of the mice. Additionally, SCG sidedness was never reported.

#### Summary

- Functional in vitro evidence indicates that the SCG indirectly contributes to cardiac innervation by its response to NGF produced by cardiomyocytes.
- Information on the relevance of SCG sidedness is unavailable in in vitro studies.
- No conclusion can be drawn with regard to sex differences as the sex is generally not reported.
- Experiments were heterogeneous in age and culture settings and were predominantly performed in postnatal tissues from the neonatal stage.

## Discussion

This systematic review evaluated current evidence for morphological and functional involvement of SCG in cardiac innervation in various species. The main findings are as follows:

1. Both morphological as well as functional evidence supports an indirect contribution of the SCG to cardiac innervation. Evidence of a direct contribution has been found morphologically but, when present, only points out a minor contribution.
2. Several gaps in current knowledge were found:
  - SCG sidedness may be relevant according to some morphological studies, but this remains uninvestigated in functional studies.
  - It is unclear whether sex differences exist, either owing to the male predominance in the studies or a lack of reporting of the sex of the included subjects.
  - It is unclear whether differences in the pre- and postnatal/adult stages exist, owing to the lack of prenatal subjects in morphological and functional in vivo studies and the heterogeneity of experiments in functional in vitro studies.

### Contribution of the SCG to cardiac innervation

In all species, morphological evidence that the SCG indirectly contributes to cardiac innervation was found, but the extent of the contribution varies per species. In humans and in primates, higher levels in the evolutionary hierarchy seem to relate to an increased prevalence of the superior cardiac nerve and increased observation of a direct connection from the superior cardiac nerve toward the heart (Fig. 4) [29]. It is unclear whether an evolutionary hierarchy exists over the entire spectrum of species, as macroscopic observation is challenging in smaller species and differential methods, such as retrograde labeling, were performed. In each case, only a few cells could retrogradely be traced back.

Functional studies revealed evidence that the SCG indirectly contributes to cardiac innervation, both in vivo and in vitro. Functional in vivo studies researching the effects of superior cervical ganglionectomy on the heart, effects of myocardial infarction on the SCG, or the effects on the heart after injecting substances into the SCG did not provide evidence for direct innervation of the heart by SCG neurons. One study investigating the direct effect by SCG stimulation failed to find a contribution [50]. These data more likely reflect indirect effects of altered sympathetic nervous system activity. Triggers, such as MI or heart failure, could lead to changes in cardiac afferent signaling, resulting in alterations in sympathetic nervous system activity through a reflex pathway rather than through a direct link [91, 92].

The majority of functional in vitro studies consisted of co-cultures of cardiac and SCG tissue or cells, cultures of SCG cells in heart-conditioned medium, or induction of MI and investigating isolated SCG neurons. Of interest, other neighboring cells of the heart and SCG, such as epicardium-derived cells or carotid body cells, may influence this interaction between SCG and cardiac innervation [65, 90]. A limitation of co-culture experiments is that a direct link between SCG and the heart cannot be unequivocally determined. These experiments rather indicate whether two cell types cultured together can influence each other. Nevertheless, these indirect results are relevant and could generate new hypotheses for future studies. Most of the included functional studies have shown that SCG neurons respond to NGF produced by cardiomyocytes. Therefore, an indirect contribution to cardiac innervation may be applicable to other sympathetic ganglia than the SCG alone, as they also respond to NGF.

In summary, the morphological data indicate a minor contribution of the SCG, whereas functional data point to a more significant contribution. This discrepancy could partly be attributed to publication bias, as negative results are rarely published and particularly functional studies are designed to test a hypothesis. This means that the relevance of the SCG in cardiac innervation may be overrepresented in the functional studies.

On the basis of the findings described above, to study the direct role of specific autonomic ganglia in cardiac sympathetic innervation, it should be considered to select sympathetic ganglia located closer to the heart, such as the stellate ganglia or cardiac ganglia. However, the SCG might still be useful to study the general role of sympathetic neurons in cardiac diseases owing to its accessibility in all species.

### Choosing the best SCG side for future studies

Approximately half of the studies performed in humans did not show any effect of sidedness, while in others the left side seemed to have a larger contribution to cardiac innervation. Morphological studies using retrograde labeling showed different results depending on the location of HRP injection. This is not surprising, because the peripheral cardiac autonomic nervous system shows considerable asymmetry, interindividual variations, and regional differences in anatomical, functional, and molecular characteristics [16]. With novel techniques such as single-cell RNA sequencing, the genetic characterization of tissues at single-cell resolution is possible [93]. However, these methods can be expensive, so best SCG side choice is critical. In this regard, functional studies could potentially contribute more as many papers included in the currently evaluated manuscripts failed to mention which side was used or whether sidedness was relevant.

### Sex differences exist in health and disease, but information in the SCG is lacking

It has become clear in recent years that marked sex differences exist in cardiac autonomic innervation [15, 94, 95]. It is important to take into account potential sex differences in SCG, as it may influence experimental outcome and shed light on sex differences in (outcomes of) cardiovascular disease. In the studies included in this systematic review, either male subjects were included or the sex was not reported at all. This is in line with our previous systematic review on hyperinnervation after myocardial infarction (manuscript under review), in which only 8% of studies specifically included the female sex. This underrepresentation of female experimental models concerns all species. Similar concerns have been addressed in human clinical studies [96].

### Prenatal stage remains underexposed

The vast majority of the studies included postnatal subjects and tissues in all three categories (morphology, functional in vivo, and functional in vitro). It is striking that the prenatal SCG has been studied to a lesser extent than postnatal/adult SCG, as prenatal SCG have the advantage of being more prone to sprout during co-culturing in vitro than adult ganglia, at least in control (“healthy”) settings. Many researchers seem to choose neonatal SCGs, which also show better sprouting capacity as compared with adult SCG, as an alternative. The controversial embryological origin of the cervical sympathetic chain ganglia may add to the preference for using postnatal/adult subjects and tissues in research. On the basis of the observation that cellular clusters will expand from the thoracic to the cervical region [97], it has been speculated that cervical ganglia are generated from the thoracic sympathetic chain [9, 98]. As there are only 3–4 cervical ganglia in the cervical region whereas at the thoracic level each spinal level has a corresponding ganglion, alternatively, it has been suggested that the development of sympathetic ganglia is associated initially with the intersegmental vessels [99]. The limited number of cervical ganglia could, in this perspective, be attributed to regression of most of the cervical intersegmental arteries with subsequent remodeling and fusion of the corresponding ganglia. The upper four cervical ganglia would thus eventually form the superior cervical sympathetic ganglion, anatomically related or induced by the developing external carotid artery [99]. Either way, changes occur during development and the contribution of SCG to the heart may vary in different stages, which may be another motivation for the choice to study postnatal/adult subjects and tissue. More information on fetal ganglia is required to comprehend the neuronal plasticity and possible re-expression of a fetal phenotype in disease states [100].

### Specific structure and function of the SCG as compared with other sympathetic chain ganglia

**Fig. 5:**
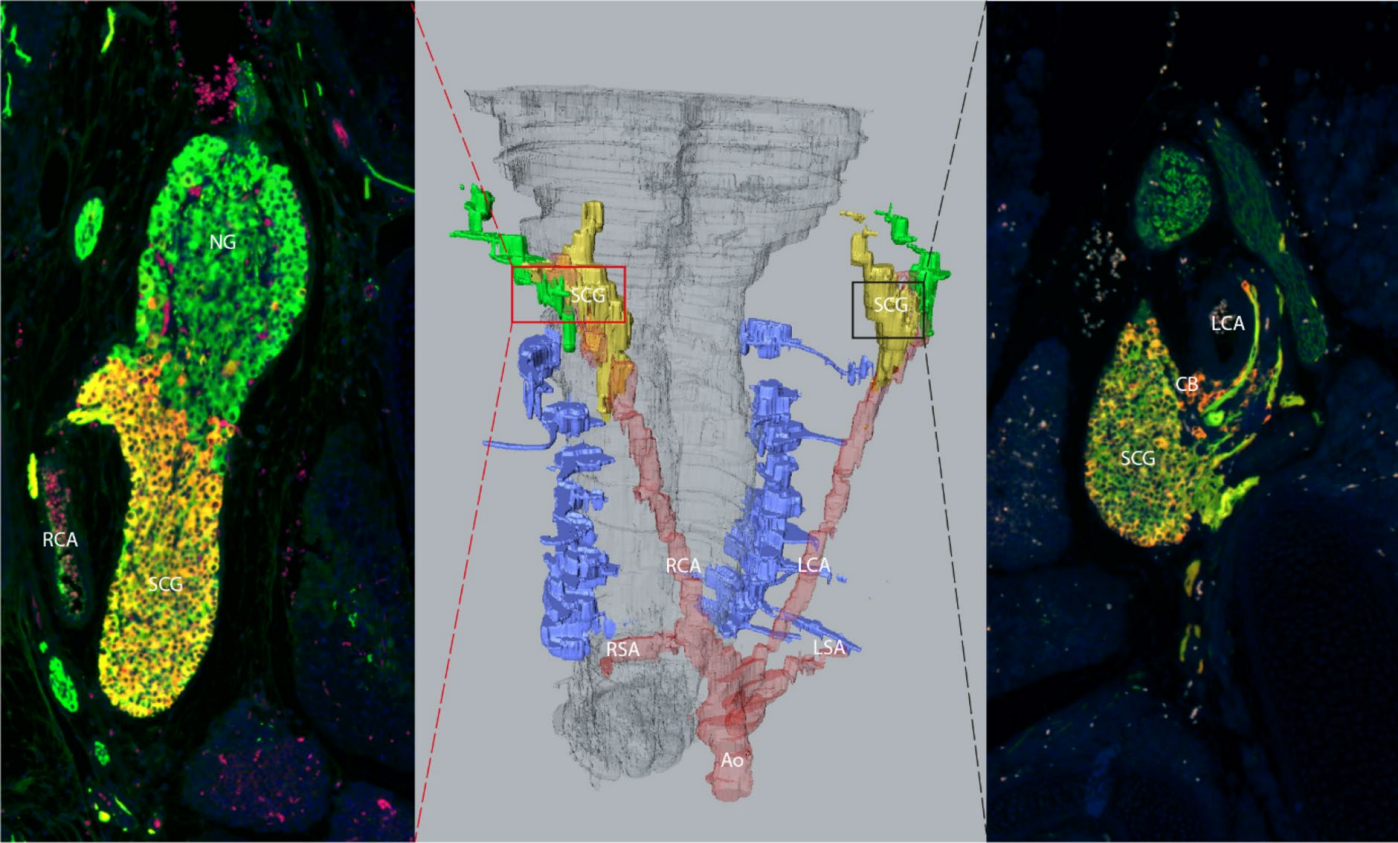
Connection of the sympathetic SCG with the nodose ganglion and carotid body in mouse. The middle panel shows a 3D reconstruction image of an embryonic female murine (18.5 days) spinal cord, carotid arteries (indicated in red), SCG (yellow), and nodose ganglion (green). *Ao* aorta, *CB* carotid body, *LCA* left carotid artery, *LSA* left subclavian artery, *NG* nodose ganglion, *RCA* right carotid artery, *RSA* right subclavian artery, *SCG*  superior cervical ganglion

The SCG is a remarkable mass of nerve cells that has a unique spatial anatomical localization (Fig. 5). It is situated between the branching point of the common carotid arteries, in close proximity to the carotid body, which it innervates [101]. The innervation pattern, however, is much broader than the cardiovascular system alone. Nerve fibers originating from the SCG provide sympathetic input toward the head, where it stimulates parts of the eye and blood vessels [102]. In this respect, it may be relevant that the SCG is situated adjacent to the above-mentioned carotid body, itself an intriguing structure involved in oxygen, carbon, and pH sensing, which has been shown to produce many neurotrophic factors [65, 103]. With regard to cardiovascular disease states, a role of the carotid body in hypertension has been indicated [104]. Although the SCG is a sympathetic chain ganglion, remarkably, a connection of the sympathetic SCG with the parasympathetic nodose ganglion has been observed in mice and rhesus monkeys [14, 32]. Therefore, a contribution or functional interaction of the SCG with cardiac parasympathetic innervation may be possible.

### Clinical relevance

Neuromodulatory interventions to treat refractory ventricular arrhythmias are emerging and include blockade or surgical removal of stellate ganglia [105]. The SCG seems to have limited contributions to cardiac innervation as compared with, e.g., the stellate ganglion, and shows important interindividual anatomical heterogeneity [14]. As the direct contribution of the SCG to cardiac innervation is likely limited and the dominant effect of the SCG appears to occur cranially, the clinical relevance seems to be limited on the basis of current knowledge. The SCG is useful to study the role of sympathetic neurons in cardiac diseases in experimental settings owing to its accessibility in all species.

### Limitations

The overall quality of the studies was moderate using our selected quality scores. The oldest included study was published in 1976, and the most recent in 2023. As many publication reporting guidelines and checklists have been developed in more recent years and journals generally have more detailed submission guidelines, the year of publication could influence the quality of the results. Additionally, using checklists, some papers may receive a higher quality than expected, as poor English and citation of faulty references might have been overlooked. By excluding non-English-language papers, some data may have been lost.

## Conclusions

Current literature supports indirect involvement of the SCG in cardiac innervation, at both a morphological and a functional level. Evidence of direct involvement seems limited. Therefore, the SCG is an adequate structure to take into account when studying the role of sympathetic structures in cardiac function in both health and disease. Studies investigating the direct contribution of sympathetic innervation to the heart should rather be focusing on the stellate or cardiac ganglia. The relevance of SCG sidedness, sex, and developmental stage in health and disease also remains unclear and warrants further exploration.

### Supplementary Information

Below is the link to the electronic supplementary material.

Supplementary file1 (DOCX 3841 KB)

## Data Availability

All data are presented in the text, figures, tables and supplementary material. No additional datasets were generated or analysed during the current study.
