# Supplementary material for: The relevance of the superior cervical ganglion for cardiac autonomic innervation in health and disease: a systematic review
Source: Clin Auton Res. 2024 Feb 23;34(1):45–77. doi: 10.1007/s10286-024-01019-2 (PMC10944423; doi:10.1007/s10286-024-01019-2)
Supplement: Supplementary file 1 — Supplementary file1 (DOCX 3841 KB) [file 10286_2024_1019_MOESM1_ESM.docx]

**Supplementary materials**

**Appendix A: Literature search query**

**Databases:**

**PubMed** Fourfold strategy:

- Superior cervical ganglion (main subject) & Heart

- Superior cervical ganglion & Heart innervation

- Superior cervical ganglion & Heart & Nerve Growth Factors

- Sympathetic ganglion & Heart & Nerve Growth Factors

**(**(("Superior Cervical Ganglion"[majr] OR "superior cervical ganglion"[ti] OR "superior cervical ganglia"[ti] OR "superior cervical gangl*"[ti] OR "ganglion cervicale superius"[ti] OR "ganglion cervical superior"[ti] OR "ganglion cervicale"[ti]) AND ("Heart"[mesh] OR "Cardiovascular System"[mesh] OR "Cardiovascular"[tw] OR "Cardiac"[tw] OR "myocardial"[tw] OR "Heart"[tw] OR "Heart Diseases"[mesh] OR "Cardiovascular Diseases"[mesh] OR "Arrhythmia"[tw] OR "Myocardial infarction"[tw] OR "cardiac overload"[tw] OR "cardiac damage"[tw] OR "heart failure"[tw] OR "cardiac damage"[tw] OR "myocardial reperfusion"[tw] OR "cardiac innervation"[tw] OR "arrhythmias"[tw] OR "atrial"[tw] OR "ventricular"[tw])) **OR** (("Superior Cervical Ganglion"[Mesh] OR "superior cervical ganglion"[tw] OR "superior cervical ganglia"[tw] OR "superior cervical gangl*"[tw] OR "ganglion cervicale superius"[tw] OR "ganglion cervical superior"[tw] OR "ganglion cervicale"[tw]) AND ("Heart/innervation"[majr])) **OR** (("Superior Cervical Ganglion"[Mesh] OR "superior cervical ganglion"[tw] OR "superior cervical ganglia"[tw] OR "superior cervical gangl*"[tw] OR "ganglion cervicale superius"[tw] OR "ganglion cervical superior"[tw] OR "ganglion cervicale"[tw]) AND ("Heart"[mesh] OR "Cardiovascular System"[mesh] OR "Cardiovascular"[tw] OR "Cardiac"[tw] OR "myocardial"[tw] OR "Heart"[tw] OR "Heart Diseases"[mesh] OR "Cardiovascular Diseases"[mesh] OR "Arrhythmia"[tw] OR "Myocardial infarction"[tw] OR "cardiac overload"[tw] OR "cardiac damage"[tw] OR "heart failure"[tw] OR "cardiac damage"[tw] OR "myocardial reperfusion"[tw] OR "cardiac innervation"[tw] OR "arrhythmias"[tw] OR "atrial"[tw] OR "ventricular"[tw]) AND ("Nerve Growth Factors"[Mesh] OR "Nerve Growth Factors"[tw] OR "Nerve Growth Factor"[tw] OR "Neurite Outgrowth Factor"[tw] OR "Neurite Outgrowth Factors"[tw] OR "Neuronal Growth Associated Protein"[tw] OR "Neuronal Growth Associated Proteins"[tw] OR "Neuronotrophic Factor"[tw] OR "Neuronotrophic Factors"[tw] OR "Neurotrophic Factor"[tw] OR "Neurotrophic Factors"[tw] OR "Neurotrophic Protein"[tw] OR "Neurotrophic Proteins"[tw] OR "Neurotrophin"[tw] OR "Neurotrophins"[tw] OR "Glia Maturation Factor"[tw] OR "Glia Maturation Factors"[tw] OR "Glial Cell Line-Derived Neurotrophic Factor"[tw] OR "Glial Cell Line-Derived Neurotrophic Factors"[tw] OR "Nerve Growth Factor"[tw] OR "Nerve Growth Factors"[tw] OR "Netrin"[tw] OR "Netrin-1"[tw] OR "Netrins"[tw] OR "Neuregulin"[tw] OR "Neuregulin-1"[tw] OR "Neuregulins"[tw] OR "Neurotrophin 3"[tw] OR "Neurturin"[tw] OR "Neurturins"[tw])) **OR** (("Ganglia, Sympathetic"[Mesh:noexp] OR "Sympathetic Ganglion"[tw] OR "sympathetic ganglia"[tw]) AND ("Heart"[majr] OR "Cardiovascular System"[majr] OR "Cardiovascular"[ti] OR "Cardiac"[ti] OR "myocardial"[ti] OR "Heart"[ti] OR "Heart Diseases"[majr] OR "Cardiovascular Diseases"[majr] OR "Arrhythmia"[ti] OR "Myocardial infarction"[ti] OR "cardiac overload"[ti] OR "cardiac damage"[ti] OR "heart failure"[ti] OR "cardiac damage"[ti] OR "myocardial reperfusion"[ti] OR "cardiac innervation"[ti] OR "arrhythmias"[ti] OR "atrial"[ti] OR "ventricular"[ti]) AND ("Nerve Growth Factors"[Mesh] OR "Nerve Growth Factors"[tw] OR "Nerve Growth Factor"[tw] OR "Neurite Outgrowth Factor"[tw] OR "Neurite Outgrowth Factors"[tw] OR "Neuronal Growth Associated Protein"[tw] OR "Neuronal Growth Associated Proteins"[tw] OR "Neuronotrophic Factor"[tw] OR "Neuronotrophic Factors"[tw] OR "Neurotrophic Factor"[tw] OR "Neurotrophic Factors"[tw] OR "Neurotrophic Protein"[tw] OR "Neurotrophic Proteins"[tw] OR "Neurotrophin"[tw] OR "Neurotrophins"[tw] OR "Glia Maturation Factor"[tw] OR "Glia Maturation Factors"[tw] OR "Glial Cell Line-Derived Neurotrophic Factor"[tw] OR "Glial Cell Line-Derived Neurotrophic Factors"[tw] OR "Nerve Growth Factor"[tw] OR "Nerve Growth Factors"[tw] OR "Netrin"[tw] OR "Netrin-1"[tw] OR "Netrins"[tw] OR "Neuregulin"[tw] OR "Neuregulin-1"[tw] OR "Neuregulins"[tw] OR "Neurotrophin 3"[tw] OR "Neurturin"[tw] OR "Neurturins"[tw]))**)**

**Embase (OVID version)**

Fourfold strategy:

- Superior cervical ganglion (main subject) & Heart

- Superior cervical ganglion & Heart innervation

- Superior cervical ganglion & Heart & Nerve Growth Factors

- Sympathetic ganglion & Heart & Nerve Growth Factors

**(**((*"Superior Cervical Ganglion"/ OR "superior cervical ganglion".ti OR "superior cervical ganglia".ti OR "superior cervical gangl*".ti OR "ganglion cervicale superius".ti OR "ganglion cervical superior".ti OR "ganglion cervicale".ti) AND (exp *"Heart"/ OR exp *"Cardiovascular System"/ OR "Cardiovascular".ti,ab OR "Cardiac".ti,ab OR "myocardial".ti,ab OR "Heart".ti,ab OR exp *"Heart Disease"/ OR exp *"Cardiovascular Disease"/ OR "Arrhythmia".ti,ab OR "Myocardial infarction".ti,ab OR "cardiac overload".ti,ab OR "cardiac damage".ti,ab OR "heart failure".ti,ab OR "cardiac damage".ti,ab OR "myocardial reperfusion".ti,ab OR "cardiac innervation".ti,ab OR "arrhythmias".ti,ab OR "atrial".ti,ab OR "ventricular".ti,ab)) **OR** ((*"Superior Cervical Ganglion"/ OR "superior cervical ganglion".ti,ab OR "superior cervical ganglia".ti,ab OR "superior cervical gangl*".ti,ab OR "ganglion cervicale superius".ti,ab OR "ganglion cervical superior".ti,ab OR "ganglion cervicale".ti,ab) AND (exp *"Heart"/ OR exp *"Cardiovascular System"/ OR "Cardiovascular".ti,ab OR "Cardiac".ti,ab OR "myocardial".ti,ab OR "Heart".ti,ab OR exp *"Heart Disease"/ OR exp *"Cardiovascular Disease"/ OR "Arrhythmia".ti,ab OR "Myocardial infarction".ti,ab OR "cardiac overload".ti,ab OR "cardiac damage".ti,ab OR "heart failure".ti,ab OR "cardiac damage".ti,ab OR "myocardial reperfusion".ti,ab OR "cardiac innervation".ti,ab OR "arrhythmias".ti,ab OR "atrial".ti,ab OR "ventricular".ti,ab) AND (exp *"neurotrophic factor"/ OR *"Nerve Growth Factor"/ OR "Nerve Growth Factors".ti,ab OR "Nerve Growth Factor".ti,ab OR "Neurite Outgrowth Factor".ti,ab OR "Neurite Outgrowth Factors".ti,ab OR "Neuronal Growth Associated Protein".ti,ab OR "Neuronal Growth Associated Proteins".ti,ab OR "Neuronotrophic Factor".ti,ab OR "Neuronotrophic Factors".ti,ab OR "Neurotrophic Factor".ti,ab OR "Neurotrophic Factors".ti,ab OR "Neurotrophic Protein".ti,ab OR "Neurotrophic Proteins".ti,ab OR "Neurotrophin".ti,ab OR "Neurotrophins".ti,ab OR "Glia Maturation Factor".ti,ab OR "Glia Maturation Factors".ti,ab OR "Glial Cell Line-Derived Neurotrophic Factor".ti,ab OR "Glial Cell Line-Derived Neurotrophic Factors".ti,ab OR "Nerve Growth Factor".ti,ab OR "Nerve Growth Factors".ti,ab OR "Netrin".ti,ab OR "Netrin-1".ti,ab OR "Netrins".ti,ab OR "Neuregulin".ti,ab OR "Neuregulin-1".ti,ab OR "Neuregulins".ti,ab OR "Neurotrophin 3".ti,ab OR "Neurturin".ti,ab OR "Neurturins".ti,ab)) **OR** ((*"sympathetic ganglion"/ OR "Sympathetic Ganglion".ti,ab OR "sympathetic ganglia".ti,ab) AND (exp *"Heart"/ OR exp *"Cardiovascular System"/ OR "Cardiovascular".ti OR "Cardiac".ti OR "myocardial".ti OR "Heart".ti OR exp *"Heart Disease"/ OR exp *"Cardiovascular Disease"/ OR "Arrhythmia".ti OR "Myocardial infarction".ti OR "cardiac overload".ti OR "cardiac damage".ti OR "heart failure".ti OR "cardiac damage".ti OR "myocardial reperfusion".ti OR "cardiac innervation".ti OR "arrhythmias".ti OR "atrial".ti OR "ventricular".ti) AND (exp *"neurotrophic factor"/ OR *"Nerve Growth Factor"/ OR "Nerve Growth Factors".ti,ab OR "Nerve Growth Factor".ti,ab OR "Neurite Outgrowth Factor".ti,ab OR "Neurite Outgrowth Factors".ti,ab OR "Neuronal Growth Associated Protein".ti,ab OR "Neuronal Growth Associated Proteins".ti,ab OR "Neuronotrophic Factor".ti,ab OR "Neuronotrophic Factors".ti,ab OR "Neurotrophic Factor".ti,ab OR "Neurotrophic Factors".ti,ab OR "Neurotrophic Protein".ti,ab OR "Neurotrophic Proteins".ti,ab OR "Neurotrophin".ti,ab OR "Neurotrophins".ti,ab OR "Glia Maturation Factor".ti,ab OR "Glia Maturation Factors".ti,ab OR "Glial Cell Line-Derived Neurotrophic Factor".ti,ab OR "Glial Cell Line-Derived Neurotrophic Factors".ti,ab OR "Nerve Growth Factor".ti,ab OR "Nerve Growth Factors".ti,ab OR "Netrin".ti,ab OR "Netrin-1".ti,ab OR "Netrins".ti,ab OR "Neuregulin".ti,ab OR "Neuregulin-1".ti,ab OR "Neuregulins".ti,ab OR "Neurotrophin 3".ti,ab OR "Neurturin".ti,ab OR "Neurturins".ti,ab))**)** NOT (conference review or conference abstract).pt

**Web of Science**

Triple strategy:

- Superior cervical ganglion (main subject) & Heart

- Superior cervical ganglion & Heart innervation

- Superior cervical ganglion & Heart & Nerve Growth Factors

**(**(ti=("Superior Cervical Ganglion" OR "superior cervical ganglion" OR "superior cervical ganglia" OR "superior cervical gangl*" OR "ganglion cervicale superius" OR "ganglion cervical superior" OR "ganglion cervicale") AND (ti=("Heart" OR "Cardiovascular System" OR "Cardiovascular" OR "Cardiac" OR "myocardial" OR "Heart" OR "Heart Disease" OR "Cardiovascular Disease" OR "Arrhythmia" OR "Myocardial infarction" OR "cardiac overload" OR "cardiac damage" OR "heart failure" OR "cardiac damage" OR "myocardial reperfusion" OR "cardiac innervation" OR "arrhythmias" OR "atrial" OR "ventricular") OR ab=("Heart" OR "Cardiovascular System" OR "Cardiovascular" OR "Cardiac" OR "myocardial" OR "Heart" OR "Heart Disease" OR "Cardiovascular Disease" OR "Arrhythmia" OR "Myocardial infarction" OR "cardiac overload" OR "cardiac damage" OR "heart failure" OR "cardiac damage" OR "myocardial reperfusion" OR "cardiac innervation" OR "arrhythmias" OR "atrial" OR "ventricular"))) **OR** ((ti=("Superior Cervical Ganglion" OR "superior cervical ganglion" OR "superior cervical ganglia" OR "superior cervical gangl*" OR "ganglion cervicale superius" OR "ganglion cervical superior" OR "ganglion cervicale") OR ab=("Superior Cervical Ganglion" OR "superior cervical ganglion" OR "superior cervical ganglia" OR "superior cervical gangl*" OR "ganglion cervicale superius" OR "ganglion cervical superior" OR "ganglion cervicale")) AND (ti=("Heart" OR "Cardiovascular System" OR "Cardiovascular" OR "Cardiac" OR "myocardial" OR "Heart" OR "Heart Disease" OR "Cardiovascular Disease" OR "Arrhythmia" OR "Myocardial infarction" OR "cardiac overload" OR "cardiac damage" OR "heart failure" OR "cardiac damage" OR "myocardial reperfusion" OR "cardiac innervation" OR "arrhythmias" OR "atrial" OR "ventricular") OR ab=("Heart" OR "Cardiovascular System" OR "Cardiovascular" OR "Cardiac" OR "myocardial" OR "Heart" OR "Heart Disease" OR "Cardiovascular Disease" OR "Arrhythmia" OR "Myocardial infarction" OR "cardiac overload" OR "cardiac damage" OR "heart failure" OR "cardiac damage" OR "myocardial reperfusion" OR "cardiac innervation" OR "arrhythmias" OR "atrial" OR "ventricular")) AND (ti=("neurotrophic factor" OR "Nerve Growth Factor" OR "Nerve Growth Factors" OR "Nerve Growth Factor" OR "Neurite Outgrowth Factor" OR "Neurite Outgrowth Factors" OR "Neuronal Growth Associated Protein" OR "Neuronal Growth Associated Proteins" OR "Neuronotrophic Factor" OR "Neuronotrophic Factors" OR "Neurotrophic Factor" OR "Neurotrophic Factors" OR "Neurotrophic Protein" OR "Neurotrophic Proteins" OR "Neurotrophin" OR "Neurotrophins" OR "Glia Maturation Factor" OR "Glia Maturation Factors" OR "Glial Cell Line-Derived Neurotrophic Factor" OR "Glial Cell Line-Derived Neurotrophic Factors" OR "Nerve Growth Factor" OR "Nerve Growth Factors" OR "Netrin" OR "Netrin-1" OR "Netrins" OR "Neuregulin" OR "Neuregulin-1" OR "Neuregulins" OR "Neurotrophin 3" OR "Neurturin" OR "Neurturins") OR ab=("neurotrophic factor" OR "Nerve Growth Factor" OR "Nerve Growth Factors" OR "Nerve Growth Factor" OR "Neurite Outgrowth Factor" OR "Neurite Outgrowth Factors" OR "Neuronal Growth Associated Protein" OR "Neuronal Growth Associated Proteins" OR "Neuronotrophic Factor" OR "Neuronotrophic Factors" OR "Neurotrophic Factor" OR "Neurotrophic Factors" OR "Neurotrophic Protein" OR "Neurotrophic Proteins" OR "Neurotrophin" OR "Neurotrophins" OR "Glia Maturation Factor" OR "Glia Maturation Factors" OR "Glial Cell Line-Derived Neurotrophic Factor" OR "Glial Cell Line-Derived Neurotrophic Factors" OR "Nerve Growth Factor" OR "Nerve Growth Factors" OR "Netrin" OR "Netrin-1" OR "Netrins" OR "Neuregulin" OR "Neuregulin-1" OR "Neuregulins" OR "Neurotrophin 3" OR "Neurturin" OR "Neurturins"))) **OR** ((TI=("Sympathetic Ganglion" OR "sympathetic ganglia") OR AB=("Sympathetic Ganglion" OR "sympathetic ganglia")) AND TI=("Heart" OR "Cardiovascular System" OR "Cardiovascular" OR "Cardiac" OR "myocardial" OR "Heart" OR "Heart Disease" OR "Cardiovascular Disease" OR "Arrhythmia" OR "Myocardial infarction" OR "cardiac overload" OR "cardiac damage" OR "heart failure" OR "cardiac damage" OR "myocardial reperfusion" OR "cardiac innervation" OR "arrhythmias" OR "atrial" OR "ventricular") AND (TI=("neurotrophic factor" OR "Nerve Growth Factor" OR "Nerve Growth Factors" OR "Nerve Growth Factor" OR "Neurite Outgrowth Factor" OR "Neurite Outgrowth Factors" OR "Neuronal Growth Associated Protein" OR "Neuronal Growth Associated Proteins" OR "Neuronotrophic Factor" OR "Neuronotrophic Factors" OR "Neurotrophic Factor" OR "Neurotrophic Factors" OR "Neurotrophic Protein" OR "Neurotrophic Proteins" OR "Neurotrophin" OR "Neurotrophins" OR "Glia Maturation Factor" OR "Glia Maturation Factors" OR "Glial Cell Line-Derived Neurotrophic Factor" OR "Glial Cell Line-Derived Neurotrophic Factors" OR "Nerve Growth Factor" OR "Nerve Growth Factors" OR "Netrin" OR "Netrin-1" OR "Netrins" OR "Neuregulin" OR "Neuregulin-1" OR "Neuregulins" OR "Neurotrophin 3" OR "Neurturin" OR "Neurturins") OR AB=("neurotrophic factor" OR "Nerve Growth Factor" OR "Nerve Growth Factors" OR "Nerve Growth Factor" OR "Neurite Outgrowth Factor" OR "Neurite Outgrowth Factors" OR "Neuronal Growth Associated Protein" OR "Neuronal Growth Associated Proteins" OR "Neuronotrophic Factor" OR "Neuronotrophic Factors" OR "Neurotrophic Factor" OR "Neurotrophic Factors" OR "Neurotrophic Protein" OR "Neurotrophic Proteins" OR "Neurotrophin" OR "Neurotrophins" OR "Glia Maturation Factor" OR "Glia Maturation Factors" OR "Glial Cell Line-Derived Neurotrophic Factor" OR "Glial Cell Line-Derived Neurotrophic Factors" OR "Nerve Growth Factor" OR "Nerve Growth Factors" OR "Netrin" OR "Netrin-1" OR "Netrins" OR "Neuregulin" OR "Neuregulin-1" OR "Neuregulins" OR "Neurotrophin 3" OR "Neurturin" OR "Neurturins")))**)** NOT dt=(meeting abstract)

**Cochrane Library**

Single strategy:

- Superior cervical ganglion & Heart

(("Superior Cervical Ganglion" OR "superior cervical ganglion" OR "superior cervical ganglia" OR "superior cervical gangl*" OR "ganglion cervicale superius" OR "ganglion cervical superior" OR "ganglion cervicale") AND ("Heart" OR "Cardiovascular System" OR "Cardiovascular" OR "Cardiac" OR "myocardial" OR "Heart" OR "Heart Disease" OR "Cardiovascular Disease" OR "Arrhythmia" OR "Myocardial infarction" OR "cardiac overload" OR "cardiac damage" OR "heart failure" OR "cardiac damage" OR "myocardial reperfusion" OR "cardiac innervation" OR "arrhythmias" OR "atrial" OR "ventricular")):ti,ab,kw

**Appendix B: Adapted version ARRIVE guidelines 2.0 used for assessment of methodological quality of in vitro studies**

| **Based on in vivo part** | 1 | In vivo study design | For each experiment, provide brief details of the animal study design including:   1. The groups being compared, including control groups. If no control group has been used, the rationale should be stated. 2. The experimental unit (e.g. a single animal, litter or cage of animals). |
| --- | --- | --- | --- |
|  | 2 | Sample size | 1. Specify the exact number of animals allocated to each group and the total number in each experiment. 2. Explain how the sample size was decided. Provide details of any a priori sample size. |
|  | 3 | Inclusion & exclusion | 1. Describe any criteria used for including and excluding animals during the experiment, and data points during the analysis. Specify if these criteria were established a priori. If no criteria were set, state this explicitly. 2. For each experimental group, report any animals, experimental units or data points not included in the analysis and explain why. If there were no exclusions, state so. 3. For each analysis, report the exact value of n in each experimental group. |
|  | 4 | Randomisation | 1. State whether randomisation was used to allocate experimental units to control and treatment groups. If done, provide the method used to generate the randomisation sequence. 2. Describe the strategy used to minimise potential confounders such as the order of treatments and measurements, or animal/cage location. If confounders were not controlled, state this explicitly. |
|  | 5 | Blinding | Describe who was aware of the group allocation at the different stages of the experiment (during the allocation, the conduct of the experiment, the outcome assessment, and the data analysis). |
|  | 6 | Experimental animals | 1. Provide species-appropriate details of the animals used, including species, (sub)strain, sex, age ( or weight) and developmental stage. 2. Provide further relevant information on the provenance of animals, health/immune status, genetic modification status, genotype, and any previous procedures |
|  | 7 | Experimental procedures | For each experimental group, including controls, describe the procedures in enough detail to allow others to replicate them, including:   1. What was done, how it was done and what was used. 2. When and how often. 3. Where (including detail of any acclimatisation periods). 4. Why (provide rationale for procedures). |
|  | 8 | Ethical statement | Provide the name of the ethical review committee or equivalent that has approved the use of animals in this study, and any relevant licence or protocol numbers (if applicable). If ethical approval was not sought or granted, provide a justification. |
|  | 9 | Housing and husbandry | Provide details of housing and husbandry conditions, including any environmental enrichment. |
|  | 10 | Animal care and monitoring | 1. Describe any interventions or steps taken in the experimental protocols to reduce pain, suffering and distress. 2. Report any expected or unexpected adverse events. 3. Describe the humane endpoints established for the study, the signs that were monitored and the frequency of monitoring. If the study did not have humane endpoints, state this. |
| **Based on in vitro part** | 11 | In vitro study design | For each experiment, provide brief details of study design including:   1. The groups being compared, including control groups. If no control group has been used, the rationale should be stated. |
|  | 12 | Sample size | 1. Specify the total number in each experiment. 2. Explain how the sample size was decided. Provide details of any a priori sample size. |
|  | 13 | Inclusion & exclusion | 1. Describe any criteria used for including and excluding data points during the analysis. Specify if these criteria were established a priori. If no criteria were set, state this explicitly. 2. For each experimental group, report any data points not included in the analysis and explain why. If there were no exclusions, state so. 3. For each analysis, report the exact value of n in each experimental group. |
|  | 14 | Randomisation | 1. State whether randomisation was used to allocate experimental units to control and treatment groups. If done, provide the method used to generate the randomisation sequence. 2. Describe the strategy used to minimise potential confounders such as the order of treatments and measurements. If confounders were not controlled, state this explicitly. |
|  | 15 | Blinding | Describe who was aware of the experimental allocation at the different stages of the experiment (during the allocation, the conduct of the experiment, the outcome assessment, and the data analysis). |
|  | 16 | Outcome measures | 1. Clearly define all outcome measures assessed (e.g. cell death, molecular markers or morphological/phenotypical changes) 2. For hypothesis-testing studies, specify the primary outcome measures, i.e. the outcome measure that was used to determine the sample size |
|  | 17 | Statistical methods | 1. Provide details of the statistical methods used for each analysis, including software used. 2. Describe any methods used to assess whether the data met the assumptions of the statistical approach and what was done if the assumptions were not met. |
|  | 18 | Experimental procedures | For each experimental group, including controls, describe the procedures in enough detail to allow others to replicate them, including:   1. What was done, how it was done and what was used (e.g. timeline, solutions, passage number, different conditions). 2. When and how often. 3. Where. 4. Why (provide rationale for the experiments). |
|  | 19 | Results | For each experiment conducted, including independent replications, report:   1. Summary/descriptive statistics for each experimental group, with a measure of variability where applicable (e.g. mean and SD or median and range) 2. If applicable, the effect size with a confidence interval |
|  | 20 | Culture conditions | Provide details for the experimental setting (e.g. medium, pH, temperature, humidity) |
| **General** | 21 | Abstract | Provide an accurate summary of the research objectives, animal species, strain and sex, cells that were used, key methods, principal findings, and study conclusions |
|  | 22 | Background | 1. Include sufficient scientific background to understand the rationale and context for the study and explain the experimental approach. 2. Explain how the animal species/model or culture conditions used address the scientific objectives and, where appropriate, the relevance to human biology. |
|  | 23 | Objectives | Clearly describe the research question, research objectives and, where appropriate, specific hypotheses being tested. |
|  | 24 | Interpretation/  scientific implications | 1. Interpret the results, taking into account the study objectives and hypotheses, current theory and other relevant studies in the literature. 2. Comment on the study limitations including potential sources of bias, limitations of the in vitro/in vivo model, and imprecision associated with the results. |
|  | 25 | Generalisability/  translation | Comment on whether, and how, the findings of this study are likely to generalise to other species or experimental conditions, including any relevance to human biology (where appropriate). |
|  | 26 | Protocol registration | Provide a statement indicating whether a protocol (including the research question, key design features and analysis plan) was prepared before the study, and if and where this protocol was registered. |
|  | 27 | Data access | Provide a statement describing if and where study data are available. |
|  | 28 | Declaration of interests | 1. Declare any potential conflicts of interest, including financial and non-financial. If none exist, this should be stated. 2. List all funding sources (including grant identifier) and the role of the funder(s) in the design, analysis and reporting of the study. |

*Table showing the ARRIVE guidelines 2.0 adjusted to in vitro studies. As all in vitro studies use biological tissue and therefore include an in vivo part of the paper, the checklist is extended and divided into an in vivo, in vitro and general part*

**Appendix C: Detailed overview of the results from the quality assessment**

**
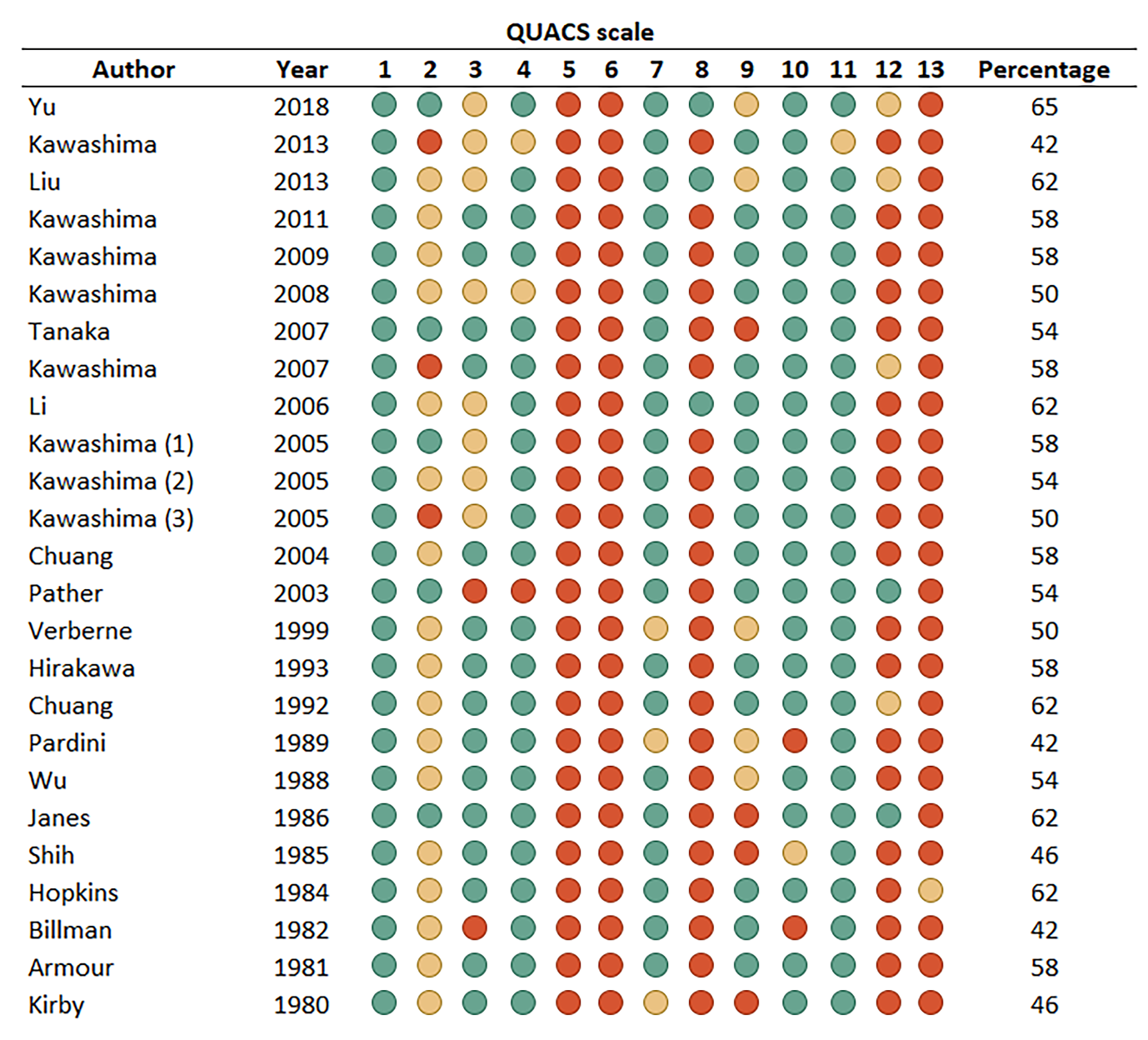
**


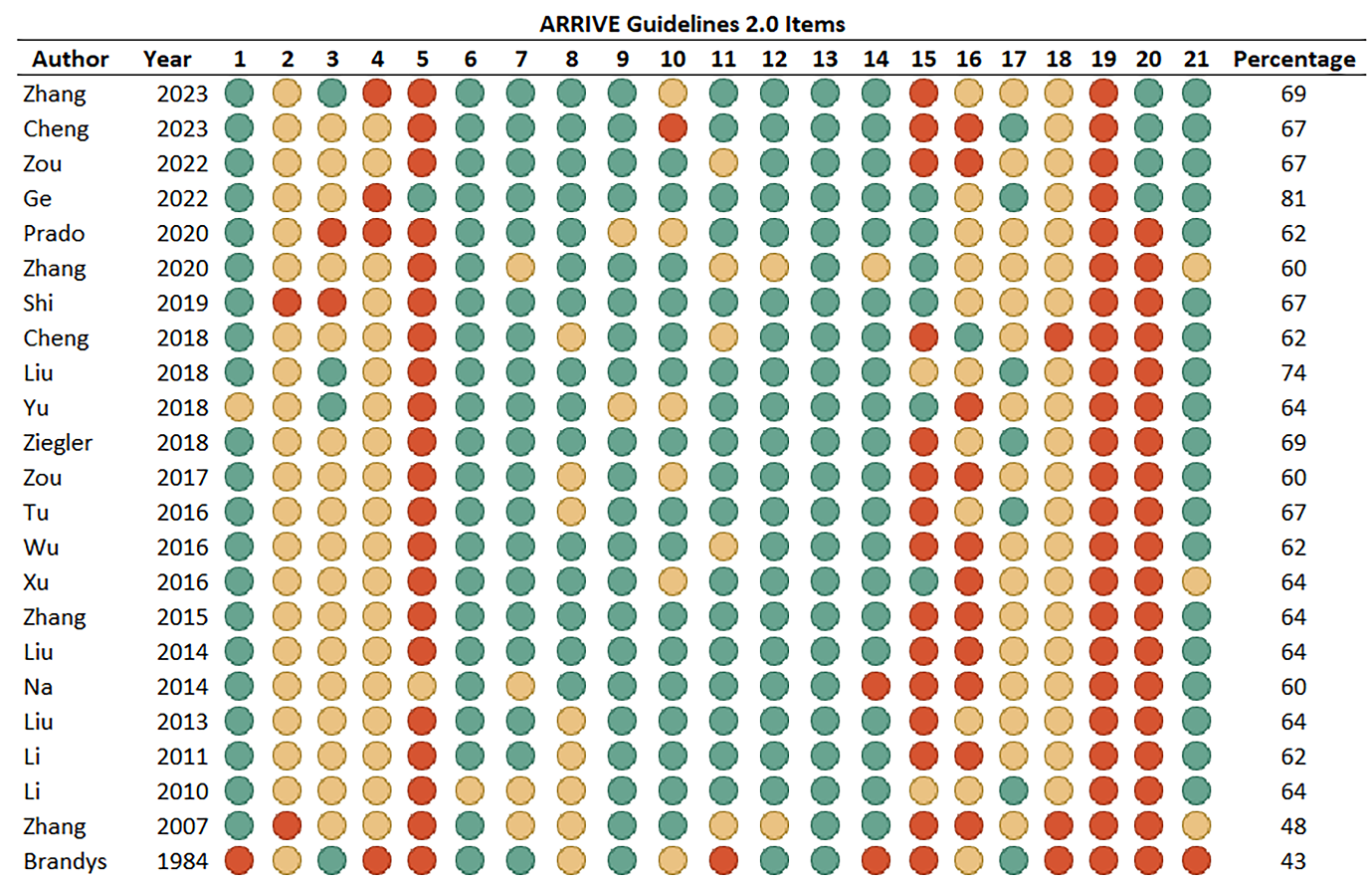


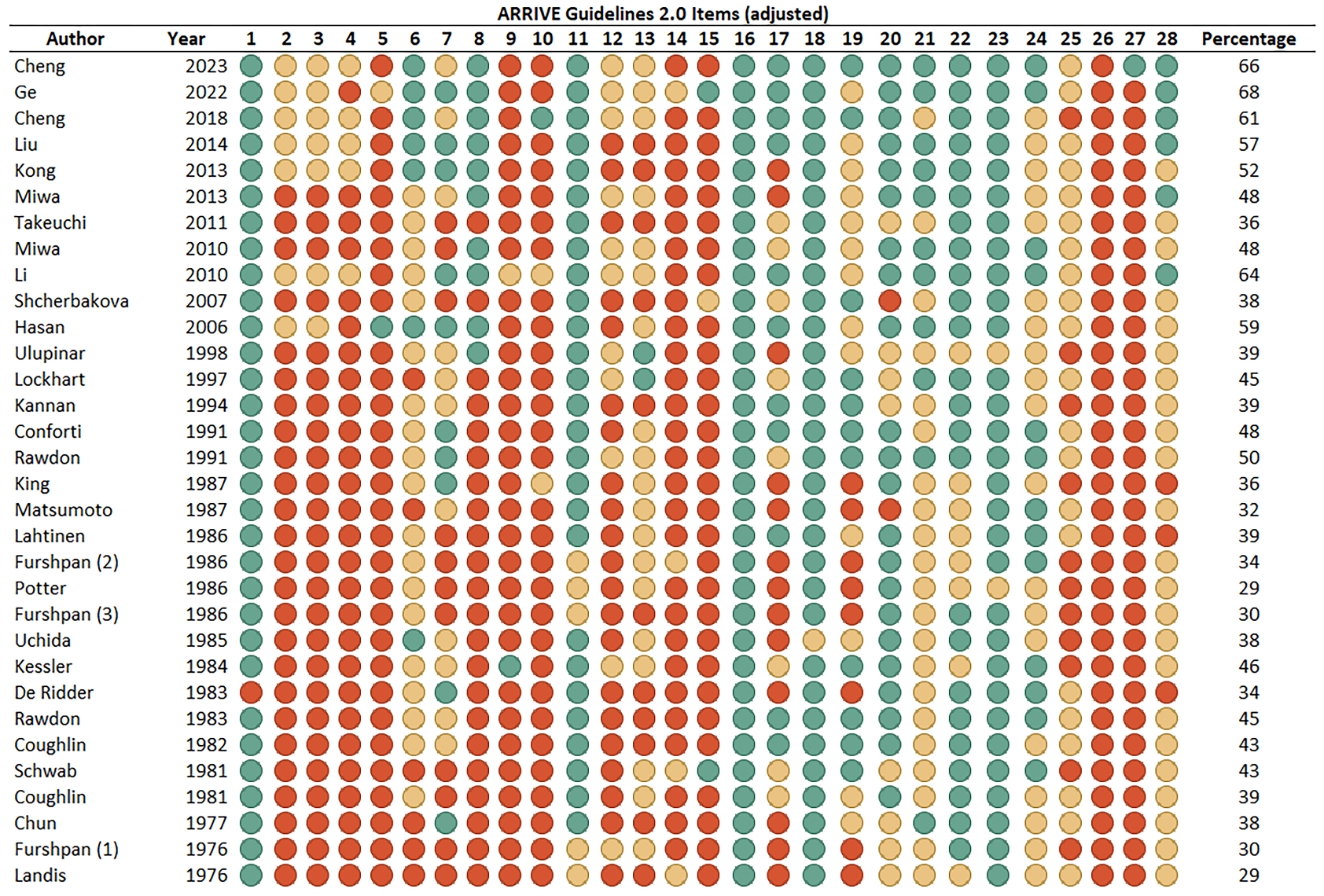


**Appendix D: Objectives and outcomes morphology studies**

| **Morphology** | | |  |  |  | |
| --- | --- | --- | --- | --- | --- | --- |
| **Author** | **Year** | **Objectives** | | | | **Outcome** |
| ***HUMAN*** |  |  | | | |  |
| Janes et al | 1986 | To describe the anatomy of the extrinsic cardiac nerves and ganglia | | | | No cardiopulmonary nerves arising from the SCG |
| Pather et al | 2003 | To determine the cervical and thoracic sympathetic contributions to the cardiac plexus | | | | 100% incidence of SCG in both fetal and adult cases  All sympathetic contributions from the cervical sympathetic trunks were found to arborize directly in the deep cardiac plexus |
| Kawashima and Sasaki | 2005 | To investigate the topological changes of the human autonomic cardiac nervous system in retroesophageal right subclavian artery (a branchial arterial anomaly) compared to the normal autonomic cardiac nervous system | | | | Case 1: the superior cardiac nerves arising from both the SCG and sympathetic trunk between SCG and MCG were observed on the right side, not on the left side  Case 2: the superior cardiac nerves arising from the SCG and sympathetic trunk between SCG and MCG were observed on both sides |
| Kawashima | 2005 | To clarify the detailed morphology of the entire autonomic cardiac nerves, including both sympathetic and parasympathetic nerves | | | | The SCG was observed in all cases  The superior cardiac nerve originating from the SCG was observed in 88.9% (15 right, 17 left)  The superior cardiac nerve originating from the sympathetic trunk between the SCG and MCG was observed in 69.4% (11 right, 14 left) |
| Kawashima et al | 2007 | To describe spatial change in the cardiac autonomic nervous system in an individual with an anomalous left vertebral artery  To compare this finding with previous reports on CANS in individuals with a retroesophageal right subclavian artery  To consider the relationship between CANS and the surrounding arterial system from this combined novel viewpoint | | | | The superior cardiac nerve in cadavers with normal arterial branching pattern arised from the SCG in 96% on the left and 81% on the right side  The superior cardiac nerve in an individual with anomalous origin of the left vertebral artery arised from the SCG in both left and right sides |
| ***NON-HUMAN PRIMATE*** |  |  | | | |  |
| Billman et al | 1982 | To present a detailed description of the cervical and upper thoracic ANS anatomy | | | | No branches from the SCG to the heart |
| Chuang et al | 1992 | To study the postganglionic neurons innervating the cardiac coronary artery | | | | After application of HRP to the main trunk of the left coronary artery 98.3% labeled neurons were localized in the SCG (the right SCG (57.4%) appeared to be more densely labeled than the left (44.6%))  After application of HRP to the main trunk of the right coronary artery 97.1% labeled neurons were found in the SCG (the right SCG (61%) appeared more densely labeled than the left (36%))  After application of HRP to the terminal branch of the ventral descending vessel of the left coronary artery 62.6% labeled neurons were observed in the right and 36.1% in the left SCG  After application of HRP to the terminal branch of the dorsal descending vessel of the left coronary artery, 96.2% labeled neurons were found in the SCG on both sides (the left SCG (54.6%) appeared more densely labeled than the right (41.6%))  There were no HRP-labeled neurons in the SCG after application to the internal surface of the pericardial sac |
| Chuang et al | 2004 | To investigate the autonomic neurons innervating the heart | | | | After HRP application in the apex of the heart the right SCG appeared more heavily labeled (63.1%) than the left (36.3%)  After application of HRP in the RV a majority of labeled cells were located in the right SCG (64.4%) and fewer in the left SCG (35.6%)  After HRP application in the sinoatrial nodal region, labeled neurons were found to be most numerous (66.8%) in the right SCG and fewer in the left SCG (33.2%)  After HRP application in the LV, labeled cells were found to be most numerous (51.1%) in the left SCG, fewer were discovered in the right SCG (38.7%)  There were no HRP-labeled cells found after injection in the pericardiac sac |
| Kawashima et al | 2005 | To clarify the general morphology of the autonomic cardiac nervous system | | | | The superior cardiac nerve never originated from the SCG, but from the sympathetic trunk between the SCG and MCG |
| Kawashima et al | 2008 | To study when the vertebral ganglion consistently appears in primate evolution  To study how the composition of the cervicothoracic ganglion is associated with primate evolution  To study when the MCG developed the communicating branches with the spinal cervical nerves  To study when the superior cardiac nerve originating from the SCG and thoracic cardiac nerve appear consistently | | | | The superior cardiac nerve originated from the SCG in 65% (4 rights, 9 left)  The superior cardiac nerve originated from the sympathetic trunk in 50% (5 right, 5 left) |
| Kawashima et al | 2009 | To report on the anatomy of the autonomic cardiac nervous system | | | | SCG is consistently present  The superior cardiac nerve never originated from the SCG, but from the sympathetic trunk between the SCG and MCG in 41.7% |
| Kawashima and Thorington | 2011 | To describe the detailed systematic morphology of the autonomic cardiac nervous system  To update and provide additional information on the surrounding nervous system  To examine the intraspecific and interspecific variation in the autonomic nervous system  To determine whether family-dependent morphology exists  To examine the relationship between the autonomic cardiac nervous system and its surrounding structures  To consider the common morphology from an evolutionary perspective within the lineage | | | | SCG is consistently present  The superior cardiac nerve never originated from the SCG, but from the sympathetic trunk between the SCG and MCG in 21.4% |
| Kawashima et al | 2013 | To elucidate the general morphology of and variations in the extrinsic cardiac nervous system  To test the hypothesis that the morphology of the cardiac innervation is a conservative structure preserving its phylogeny  To consider whether the tarsier's morphology is more similar to that of the strepsirrhini or new world monkeys  To update and provide additional information on the relationship between the neglected cardiac nervous system and its surrounding structures | | | | The superior cardiac nerve never originated from the SCG, but from the sympathetic trunk between the SCG and MCG |
| ***DOG*** |  |  | | | |  |
| Armour and Hopkins | 1981 | To determine the locations of the cells of origin of cardiac postganglionic sympathetic fibers | | | | Occasional labeled neurons were located in the caudal regions of the ipsilateral superior cervical ganglion  No labeled neurons were observed contralaterally  No exclusive origin for any individual cardiac nerve |
| Hopkins and Armour | 1984 | To determine whether a topographical organization related to regions of the heart exists within cervicothoracic ganglia and whether specific regions of the heart with specific functions are innervated by neurons in specific locations in the ganglia | | | | Innervation of a specific region of the heart does not arise from neurons in one locus of a ganglion. Very few sympathetic cardiac postganglionic neurons are found in the superior cervical ganglia. |
| Hirakawa et al | 1993 | To determine the origin of cardiac sympathetic postganglionic fibers and to demonstrate their distribution in the heart | | | | A small number of labeled cells found in bilateral SCG after injection of tracers into various regions of the heart |
| ***CAT*** |  |  | | | |  |
| Shih et al | 1985 | To investigate whether sympathetic innervation to the heart may be more specific with respect to the ganglionic origin of postganglionic neurons than previously believed  To investigate if the sympathetic supply to the cardiac regions of the apex, to the sinoatrial nodes and to the right and left ventricles may differ from one another | | | | No HRP-positive neurons where observed in the superior cervical ganglia after injections into the apex  Occasionally labeled cells in the bilateral SCG after injection into the ventral wall of the RV  Occasionally labeled cells in the bilateral SCG after injection into the dorsal wall of the LV  Occasionally labeled cells in the right SCG after injection into the Sinoatrial nodal region  No HRP-positive neurons where observed in the superior cervical ganglia after injections into pericardial sac |
| Wu et al | 1988 | To examine the retrograde axonal origin of the postganglionic innervation of the coronary arteries  To trace the innervation of the terminal branches of the coronary arteries | | | | Few cells in the SCG after application of HRP to the main trunk of the left coronaries  Occasionally some labeled cells in the SCG after application of HRP to the main trunk of the right coronaries  Few cells in the LSCG, no neuron in the RSCG after HRP application to the terminal branch of the ventral descending vessel of the left coronary artery  No neurons in the SCG after HRP application to the terminal branch of the dorsal descending vessel of the right coronary artery or pericardial sac |
| ***GUINEA PIG*** |  |  | | | |  |
| Li et al | 2006 | To demonstrate the coexistence of histamine and norepinephrine within sympathetic neuron to reveal the modulation mechanisms of histamine release from sympathetic terminals  To reveal the modulation mechanisms of histamine release from sympathetic terminals | | | | The distribution of anterogradely traced sympathetic axons and varicosities from SCG were observed and histamine and norepinephrine were identified within the same traced axons or varicosities |
| ***RAT*** |  |  | | | |  |
| Pardini et al | 1989 | To investigate the organization of the cardiac sympathetic innervation of the rat | | | | 3% of the labelled somata were found bilaterally in the SCG after injection of the left ventricular free wall  1% of the labelled somata were found bilaterally in the SCG after injection of the right ventricular free wall |
| Liu et al | 2013 | To investigate the effects of the P2X7 receptor on the increased sympathoexcitatory action in SCG caused by MI injury  To examine if there is a specific connection between SCG and the cervical dorsal root ganglion (DRG) afferents which would exaggerate the sympathoexcitatory reflex due to activation | | | | Little HRP staining in Sham SCG, more in MI SCG |
| Yu et al | 2018 | To verify if the functional oxytonicergic receptors were co-localized with the sympathetic preganglionic neurons | | | | After WGA-HRP injection into the left ventricle wall, retrogradely labeled tracer was observed in both left and right SCG  Little HRP staining in Sham SCG, more in MI SCG |
| ***MOUSE*** |  |  | | | |  |
| Manousiouthakis et al | 2014 | To explore cardiac sympathetic axon guidance in the developing mouse with specific intention of identifying common themes used by other sympathetic nerves and unique processes that reflect the unique biology of the heart  To find whether sympathetic axons from the stellate ganglion follow veins to reach and innervate the SA node and ventricular myocardium  To demonstrate the role of vascular-derived endothelin in growth and guidance of the stellate ganglion axons to the heart | | | | A portion of the SCG nerves project caudally along the common carotid arteries towards the heart  By E16.5 these reach the aorta and pulmonary trunk and then intermix on the ventral side of the heart with the ventral projections from the stellate ganglia  The SCG nerves do not project to the dorsal side of the heart |
| ***SHREW*** |  |  | | | |  |
| Tanaka et al | 2007 | To elucidate the routes of the sympathetic cardiac nerves from their origin to the heart | | | | The nerve originating from the SCG descended to reach the aortic arch and formed nerve plexuses after reaching the arterial pole supplying nerves to the ventral wall of the ventricle |
| ***CHICK*** |  |  | | | |  |
| Kirby et al | 1980 | To correlate the developing innervation of the chick heart with the known innervation of the adult chicken heart | | | | No connections from SCG to the heart |
| Verberne et al | 1999 | To investigate in detail the contribution to cardiac innervation  To study the neural crest origin of the SCG to show their relationship with the sympathetic neural crest | | | | No direct connection between the SCG and the heart, but via the carotid nerve, which joins the nodose ganglion of the vagal nerve of which TH-positive branches enter the arterial and venous pole of the embryonic chick heart |

Only outcome data was extracted that specifically related to the SCG. MCG=middle cervical ganglion. MI=myocardial infarction.

**Appendix E: Objectives and outcomes functional in vivo studies**

| **Functional – in vivo** | | | |
| --- | --- | --- | --- |
| **Author** | **Year** | **Objectives** | **Outcome** |
| ***DOG*** |  |  |  |
| Brandys et al | 1984 | To determine whether and how the heart could be affected by activation of neurons within specific loci of sympathetic ganglia | No cardiac responses were elicited by stimulation within the SCG |
| ***RABBIT*** |  |  |  |
| Cheng et al | 2018 | To investigate the effects of ischemia and fluvastatin treatment on the ion channel characteristics of SCG neurons in a MI model | The expressions of KCNQ3 (major gene corresponding to the channel protein of the potassium channel) and SCN9A (major gene corresponding to the channel protein of sodium channel ) in the MI-7days and MI-14days groups were significantly higher than that in the control group  The expressions of SCN9A in the fluvastatin-7days and fluvastatin-14days groups were not significantly different compared with the control group  Fluvastatin partially reversed the mRNA expressions of the channel protein changes induced by MI |
| Cheng et al | 2023 | To determine the changes in the ion channel characteristics of the SCG following myocardial infarction (MI) and the role of pretreatment with the P2Y12 receptor antagonist ticagrelor (TIC). | P2Y12 receptor antagonist (TIC) pretreatment partly reversed P2Y12 expression and abnormal neuronal electrophysiological changes in SCGs after MI. |
| ***GUINEA PIG*** |  |  |  |
| Li et al | 2006 | To demonstrate the coexistence of histamine and norepinephrine within sympathetic neurons  To reveal the modulation mechanisms of histamine release from cardiac sympathetic terminals | Histamine and norepinephrine were identified in the same neurons and terminals and were both significantly attenuated after chemical sympathectomy or reserpine |
| ***RAT*** |  |  |  |
| Zhang et al | 2007 | To examine the effects of the P2X3 receptor agonist A-317491 on P2X3 expression in the SCG, in naïve rats and in a myocardial ischemic rat model | In the group of MI+A317391 the animals showed short-lasting immobilization compared to MI  Staining of the P2X3 receptor in the SCG neurons of rats with MI appeared more intense than those of control and MI+A317491 rats  P2X3 protein levels were higher in MI compared to control and MI+A317491  The level of P2X3 mRNA expression was higher in MI than control or MI+A317491 |
| Li et al | 2010 | To examine whether myocardial infarction will induce changes in systolic blood pressure, heart rate and respiration, the expression value of TH and P2X2/3 receptor in SCG and myocardial tissues, and the effects of P2X2/3 receptor antagonist A-317491 on above changes | The systolic blood pressure, heart rate and respiration in the MI rats were higher than those in control rats and were reduced in P2X2/3 receptor antagonist A-317491 treated rats  The content of ATP from myocardial ischemic group was higher than that from the control group  Rats treated with A-317491 had a lower ATP content than the ischemic rat group and no difference was observed when compared to control  Coexpression value of P2X2, P2X3 and TH in the SCG and myocardial tissues of myocardial ischemic group was increased significantly in comparison with that in control group  Coexpression value of P2X2, P2X3 and TH in the SCG and myocardial tissues of myocardial ischemic rats treated with A-317491 was lower than that in the myocardial ischemic rats |
| Li et al | 2011 | To examine whether oxymatrine modulates the increased sympathoexcitatory reflex induced by MI nociceptive signalling via P2X3 receptor in rat SCG and DRG neurons | Systolic blood pressure and heart rate in the MI rats were increased, compared with those in control rats, which was decreased after treatment with oxymatrine  Norepinephrine level was increased in MI rats, but NE level in MI + Oxy group was significantly decreased compared with those in the MI group and there was no difference between control and MI+oxy  The P2X3 receptor of MI rats appeared to be more intense than in control, the IOD was lower in MI+ oxy compared to MI and there was no difference detected in MI+oxy vs control  The mRNA expression of P2X3 in SCG was higher in the MI group when compared to control and the expression of MI+oxy was lower when compared to the MI group, there was no difference between control and MI+oxy  Protein IOD was lower in control group, when compared to the MI group, the expression value in MI group was higher than that in MI+ oxy (P<0.05) and no significant difference was found in protein levels between the control and MI+oxy group |
| Liu et al | 2013 | To investigate the effects of the P2X7 receptor on the increased sympathoexcitatory action in SCG caused by MI injury and examine if there is a specific connection between SCG and the cervical dorsal root ganglion (DRG) afferents which would exaggerate the sympathoexcitatory reflex due to activation  of P2X7 receptor during MI injury | Blood pressure and heart rate in the MI group were increased when compared to sham and con+oxATP , oxATP treatment lowered the blood pressure and heart rate after MI , but heart rate in MI+oxATP group was still higher than con+oxATP group and sham group  20 days after MI, the abnormal Q wave appeared obviously in MI rats compared with that in sham or oxATP-treated control rats. After treatment with oxATP, abnormal Q wave induced by myocardial ischemia was improved in comparison with that in MI group  Treatment with siRNA P2X7 in MI rats lowered systolic blood pressure and heart rate when compared to MI+scramble siRNA, siRNA treated mice presented an improved Qwave when compared to MI+ scramble siRNA, no significant difference was observed between sham and MI+siRNA group  Both TNF-α and IL-6 were higher in MI group than in sham group, con+oxATP group, or MI+oxATP group and there were no significant differences among the sham group, con+oxATP group, and MI+oxATP group  Serum concentrations of CK-MB, CK, LDH, and cTn-I in the MI rats were increased compared with those in the sham or con+oxATP rats, P2X7 receptor antagonist oxATP significantly attenuated the elevated serum concentrations, serum concentrations in the MI+ oxATP group were higher than those in con+oxATP group or sham group, after MI rats were treated with siRNA P2X7, serum concentrations were decreased compared to the MI group and MI+scramble siRNA group  Higher expression of P2X7 mRNA and protein occurred in MI group in comparison with sham group, con+oxATP group, and MI+ oxATP group  The xpression of P2X7 receptor in MI group was significantly higher than that in sham group, con+oxATP, and MI+oxATP (p<0.05) and no significant difference was found among sham group, con+oxATP group, and MI+oxATP group  The IOD ratio of p-ERK1/2 to total ERK1/2 in MI group was higher than that in sham group and oxATP attenuated nearly 50 % of the upregulated expression due to MI.  The double-label immunofluorescence of P2X7 and TH in MI group exhibited higher density than that in sham group, con+oxATP group, and MI+oxATP group, there was no significant difference among con+oxATP group, sham group, and MI+oxATP group  20 days after MI, the staining of GS and P2X7 by double-label immunofluorescence in the MI group was more intense than that in con+ oxATP group, sham group, and MI+oxATP group, the staining of P2X7 in MI group was more intense when compared to Sham, after siRNA P2X7 knockdown in myocardial ischemic rats, the expression levels of GS or P2X7 were lower than those in the MI group, the coexpression staining of GS and P2X7 in MI+ siRNA knockout was significantly lower than that in the MI group  The staining of neuronal nuclei and P2X3 by double-label immunofluorescence in SCG of the MI group was more intense compared to the sham, con+oxATP and MI+oxATP group  The levels of GS immunoreactivity in the MI group were higher than those in the sham and con+oxATP group and oxATP treatment could completely reverse the upregulation of GS due to MI  The MI group exhibited higher CGRP or SP immunoreactivity than the sham and con+oxATP group and oxATP treatment could abolish the induced upregulation |
| Kong et al | 2013 | To define the role of P2X7 receptor of the superior cervical ganglion neurons in rat myocardial ischemic injury and explore the mechanism of cellular signal transduction after activation of P2X7 receptor | The expression of P2X7 at mRNA level in the MI group was higher than that in the control, sham and BBG-treated MI group  The amount of P2X7 protein in the MI group was higher compared to the control, sham and BBG-treated MI group  The protein mass of p-ERK1/2 in the MI group was higher compared to the control, sham and BBG-treated MI group No significant difference in ERK1/2 expression was found |
| Liu et al | 2014 | To examine the effects of puerarin on the sympathoexcitatory response induced by myocardial ischemia and explore the relationship with P2X3 receptor in rat SCG | Systolic blood pressure and heart rate were increased in the MI group compared to control and MI+Pue; 2) TH and P2X3 receptor were co-expressed in SCG neurons and cardiac tissues and appeared more intense in the MI groupcompared to control and MI+Pue; 3) The integrated optical density of P2X3 mRNA, P2X3 immunoreactivity and P2X3 protein levels in MI group were higher than control and MI+Pue |
| Na et al | 2014 | To examine whether left SCG block can treat pulmonary arterial hypertension by increasing the availability of nitric oxide | Right ventricular systolic pressure was significantly increased after monocrotaline administration compared with the control group, whereas this change was attenuated by SGB  RV/LV ratio, which was increased 1.6× in monocrotaline-treated rats compared with control rats, was markedly reduced by treatment with SGB  The LF/HF ratio, which represents sympathovagal balance, was significantly increased in monocrotaline-treated rats compared with baseline and control group values  The monocrotaline-SGB group had a significantly lower LF/HF ratio compared with those of the monocrotaline and control groups  Plasma nitrite levels were significantly reduced in monocrotaline-treated rats compared with the control and monocrotaline-SGB groups  Monocrotaline significantly impaired superoxide dismutase activity, which was reversed in SGB-treated rats. |
| Zhang et al | 2015 | To observe the change of blood pressure, heart rate after baicalin treatment in MI, and the relationship between effects of baicalin on the sympathoexcitatory reflex and the upregulated expression of P2X3 receptor in the SCG after MI | SBP, DBP and heart rate in MI rats were increased compared to normal control  SBP, DBP and heart rate in MI rats treated with baicalin were decreased compared to MI  SBP and heart rate in MI rats treated with baicalin were slightly higher than the normal control, sham and baicalin control group  The Q wave in MI rats treated with the baicalin did not appear to be pathological  The compensatory hypertrophy, large area of connective-tissue overgrowth, and bleeding in the MI rats treated with baicalin were improved compared with in the MI group  CK-MB and cTn-T in MI rats were increased compared with normal control rats, after baicalin treatment in MI rats the serum concentration of CK-MB and cTn-T was lower than in MI rats  The IOD values of P2X3 receptor in the MI group were significantly higher the control group, after treatment with baicalin in the MI rats, the IOD values of P2X3 receptor were lower than in MI rats, the IOD values of P2X3 receptor in the MI rats treated with baicalin were higher than those in the normal control, baicalin control and sham group  The average P2X3 mRNA expression in the MI group was significantly higher than those in the normal control, sham and baicalin control group, the expression levels of P2X3 mRNA in MI rats treated with baicalin were lower than in the MI group, but were higher than in the normal control and sham group  The P2X3 protein expression in the MI group were significantly higher than those in the normal control, sham group and baicalin control group, the expression levels of P2X3 protein in MI rats treated with baicalin were lower than in the MI group (p < 0.05), but were higher than in the normal control and sham group  The epinephrine concentration in the MI group was significantly higher than in the normal control, sham and baicalin control group, the concentration of epinephrine in MI rats treated with baicalin were lower than in the MI group, no difference was found among the normal control, MI rats treated with baicalin, baicalin control and sham group  The ATP concentration in the MI group was significantly higher than in the normal control, sham and baicalin control group, the concentration of ATP in MI rats treated with baicalin were lower than in the MI group, no difference was found in the ATP concentration among the normal control, MI rats treated with baicalin, baicalin control and sham group |
| Tu et al | 2016 | To investigate the effects of a small interfering RNA (siRNA) against the NONRATT021972 lncRNA on the abnormal changes of cardiac function mediated by the upregulation of the P2X7 receptor in the SCG after MI | The expression of NONRATT021972 was higher in the MI than the control group, treatment of MI rats with NONRATT021972 siRNA reduced the expression of NONRATT021972  After 30 days, the SBP, DBP and heart rate in the MI group were increased compared to control, the SBP, DBP, and heart rate in the MI group were also increased compared to the sham, MI +BBG, MI +NONRATT021972 si, and MI + P2X7 si group, the SBP, DBP, and HR in the MI rats treated with NONRATT021972 siRNA group were decreased compared with rats in the MI group  After 30 days after MI, there was an obvious abnormal Q wave in the MI and MI + SC si groups, the abnormal changes in the ECGs in the MI rats treated with NONRATT021972 siRNA group were significantly improved compared with rats in the MI group, the abnormal changes in the ECGs resulting from MI were greatly improved after the animals were treated with BBG, or P2X7 siRNA.  The LF and HF values in the MI group were decreased compared to control, sham, MI+BBG, MI+ P2X7 si, and MI +NONRATT021972 si groups  The LF/HF ratio in the MI group was higher than in control, sham, MI+BBG, and MI+P2X7 si groups  The LF/HF ratio in the MI rats treated with NONRATT021972 siRNA was decreased compared to MI rats  The IOD of GAP43- and TH-positive fibers around the ischemic myocardium of the MI group were increased compared with control, sham, MI + BBG, MI + NONRATT021972 si, and MI+ P2X7 si groups, the TH/GAP43 ratio in the MI rats was increased compared to those in the con, sham, MI + BBG, and MI+ P2X7 si groups (P< 0.01), the TH/GAP43 ratio in the MI rats treated with NONRATT021972 siRNA group was significantly decreased compared with rats in the MI group  The serum concentrations of NE and EPI in the MI group were increased compared with the control group, sham, MI + BBG, and MI + P2X7 si groups, the serum concentrations of NE and EPI in the MI rats treated with NONRATT021972 siRNA group were significantly decreased compared with rats in the MI group  The intensity of the P2X7 mRNA transcripts in the MI group was increased compared with the control and sham group, the levels of the P2X7 mRNA transcripts in the MI rats treated with NONRATT021972 siRNA group were decreased compared with rats in the MI group, the levels of the P2X7mRNAtranscripts in the MI+ BBG, and MI+P2X7 si groups were also significantly lowered compared to the MI group  The intensity of the P2X7 immunoreactivity in the MI group was increased compared with the control, sham, MI + BBG, and MI + P2X7 si groups, the intensity of the P2X7 immunoreactivity in the MI rats treated with NONRATT021972 siRNA group were significantly decreased compared with rats in the MI group  The IOD of the P2X7 protein expression in the MI group was increased compared with the control, sham, MI+BBG, and MI+ P2X7 si groups, the IOD of the P2X7 protein in the MI rats treated with NONRATT021972 siRNA group were decreased compared with rats in the MI group  The IOD of p-ERK1/2 expression in the MI group was increased compared with the control, sham, MI+BBG, and MI+ P2X7 si -groups, the IOD of p-ERK1/2 expression in the MI rats treated withNONRATT021972 siRNA group was significantly decreased compared with rats in the MI group |
| Wu et al | 2016 | To investigate the effects of lncRNA uc.48+ siRNA on the cardiac dysfunction of type 2 diabetic rats mediated by the upregulation of P2X7 receptor in the SCG | The expression of uc.48+ in SCG was significantly higher in DM than control, treatment of DM with uc.48+siRNA decreased the expression of uc.48+ compared to DM  Heart rate, SBP, DBP and mean arterial pressure were increased in DM and DM+scramble siRNA, Uc.48+siRNA treatment significantly decreased HR, SBP, DBP and MBP in DM rats  P2X2, P2X3, P2X5, P2X7 mRNA expression was increased in DM, Uc.48+siRNA significantly decreased P2X7 expressions but not the other P2X subtypes  The ratio of p-ERK1/2 to ERK1/2 was higher in DM than control, this ratio was lower in the uc.48+siRNA group than DM |
| Xu et al | 2016 | To analyze whether the effect of NONRATT021972 siRNA on heart rate variability in diabetic rats, if possible, may be related with its action on TNFα and expression of insulin receptor substrate 1 and its serine phosphorylation in SCG | NONRATT021972 expression was significantly higher in the DM group than control and could be inhibited after treatment with NONRATT021972 siRNA  HRV measurements suggest a reduction of both sympathetic and parasympathetic tone in DM rats, LF/HF ratio was increased in DM rats, suggesting a relatively high sympathetic tone, which was alleviated after NONRATT021972 siRNA treatment  Levels of TNFα mRNA in the DM group were higher than control and the TNFα mRNA in DM+NONRATT021972siRNA group were lower than DM  Insulin receptor substrate 1 phosphorylation of serine residue is elevated in DM and reduced after NONRATT021972 siRNA treatment  Staining of insulin receptor substrate 1 and neuronal nuclei was lower in the DM group compared to control, after NONRATT021972 siRNA treatment, the co-expression staining was significantly higher than DM |
| Zou et al | 2017 | To clarify whether the P2Y12 receptor is involved in the sympathoexcitation reflex after MI | Real-time PCR showed that P2Y12 mRNA expression in the MI and MI + NC groups was significantly higher than in the sham group, no difference was found between control and sham, in MI rats treated with P2Y12 shRNA, expression levels were lower than in untreated MI rats, no difference was found between the MI and MI + NC groups  P2Y12 IOD were higher in the MI than in the sham group, no difference was found between control and sham groups, after treatment with P2Y12 shRNA, the IOD was lower in the treatment group than in untreated MI rats, no difference was found between the MI and MI + NC groups  At 14 days after MI, SBP, DBP and heart rate were higher in the MI group than in the sham group, no difference was found between control and sham groups, after treatment of MI with P2Y12 shRNA, SBP, DBP and heart rate were lower than in the MI rats, no difference was found between MI and MI + NC rats  Abnormal changes in the ECGs of MI rats treated with P2Y12 shRNA group were improved compared with the MI rats  Hematoxylin-eosin staining in MI rats revealed solidified necrosis of myocardial fibers, nuclear fragmentation and disappearance, and an irregular, coarse granular cytoplasm, myocardial fibers were pink in the control and Sham groups, and the cytoplasm and nucleus were clear, the cell membrane exhibited good integrity, and the cellular arrangement was orderly with moderate intercellular spacing, after treatment of the MI rats with P2Y12 shRNA, the solidified necrosis of myocardial fibers, as well as nuclear fragmentation and disappearance, were improved, no difference was found between the MI and the MI + NC rats  Co-expression values for P2Y12 and GFAP in the MI group were higher than those of the sham group, no difference was found between control and sham rats, coexpression values for P2Y12 and GFAP were lower in MI rats treated with P2Y12 shRNA than in untreated MI rats, there was no significant difference between the MI group and the MI + NC group  Compared with the sham group, GFAP expression levels in the MI group were significantly increased, relative expression of GFAP was lower in MI rats after treatment with P2Y12 shRNA than in untreated MI rats, there was no difference between the MI and MI + NC groups  Expression of TNF-α in the MI group was elevated compared with the sham group, no difference was found between control and sham rats, relative expression of TNF-α was lower in MI rats treated with P2Y12 shRNA compared with untreated MI rats, there was no difference between the MI group and the MI + NC group  There was no difference for IOD ratio of P38/β actin between MI and sham rats, compared with the sham group, the IOD ratio of p-P38/P38 in the MI group was significantly higher, no difference was found between control and sham rats, the IOD ratio of p-P38/P38 was lower in MI rats treated with P2Y12 shRNA relative to the untreated MI rats, there was no difference between the MI group and the MI + NC group |
| Liu et al | 2018 | To examine the effects of bilateral superior cervical sympathectomy on the progression of β-aminopropionitrile-induced aortic dissection | The incidence of aortic dissection was decreased by superior cervical gangliectomy treatment from 66.7% to 20%  Rats in the BAPN+SCGx group showed a significant decrease in heart rate compared with BAPN alone  SCGx attenuated the thickening of the aortic wall by BAPN administration  Matrix metalloproteinase-9 concentrations were reduced after SCGx |
| Yu et al | 2018 | To verify if the functional oxytonergic receptors were co-localized with the sympathetic preganglionic neurons | OTR expression is increased at 7 days postinfarction compared to SHAM  The co-expression value of OTR and tyrosine hydroxylase in the SCG was higher in the MI group compared to SHAM  After HRP injection into the cardiac apex and conus arteriosus, double immunolabelling of HRP and OTR in the SCG of SHAM group was minimal in contrast to intense double labelling in the MI group |
| Shi et al | 2019 | To explore the functional significance of SCG post MI and whether the GABAergic signal system is involved in the process. | Myocardial infarction leads to a significant upregulation of NE levels in the plasma and myocardium, significantly higher arrythmia score, RSNA and HR compared to sham. Exogenous treatment with GABA attenuated these effects. Targeted knockdown of GABAARβ2 deteriorated NE levels, RSNA and mortality rates after MI. |
| Prado et al | 2020 | To analyze the cardiac electrophysiological effects of the loss of melatonin circadian oscillation and the role played by myocardial melatonin membrane receptors, SERCA2A, TNFalpha, nitrotyrosine, TGFbeta, KATP channels and connexin 43 | Reperfusion ventricular arrhythmias increased in rat hearts isolated after 3 weeks of surgical removal of the SCG  QRS interval increased during ischemia and reached a significant difference at the first minute of reperfusion in the SCGx animals. QT interval showed more dispersion during last minutes of ischemia and beginning of reperfusion in the SCGx group, QTc from SCGx hearts shortened during the last 5 minutes of ischemia, PR interval showed more dispersion during ischemia in the SCGx group, no ECG differences in the pre-ischemic period, Heart rate decreased during ischemia and partially recovered during reperfusion in both groups; Epicardial action potential duration was longer in SCGx hearts during pre-ischemia, but this prolongation did not modify the QT interval duration in the ECG, the prolongation in APD was significant from 50% of repolarization; SCGx had a delay in action potential upstroke during ischemia and suffered more action potential shortening during the last 3 min of ischemia. Early after-depolarizations triggered PVC only in SCGx  The expression of both melatonin receptors MT1 and MT2 decreased in SCGx in cardiomyocytes but remained unchanged in the vasculature. SERCA2A declined in myocytes of SCGx  SCGx increased K-ATP channels and connexin 43 lateralization without changing TNFα or nitrotyrosine in SCGx hearts  SCGx did not increase markers of fibrosis |
| Zhang | 2020 | To investigate whether SCG neurons express SCG10 (marker of axon regeneration) and whether SCG10 is involved in sympathetic nerve remodeling after MI | TH content in the infarction border zone was significantly higher 7 days after MI than in SHAM and lower than when treated with JNK inhibitor SP600125 (JNK can phosphorylate some sites on the SCG10 domain, thus accelerating the decomposition of SCG10)  SCG10 content in the SCG and infarction border zone was significantly increased 3 days after MI and even higher after 7 days  SCG10 content in the SCG and infarction border zone was significantly lower in MI after 7 days than when treated with SP600125 |
| Zou | 2022 | To explore the potential role of lncRNA uc.48+ in the SCGs in the upregulated P2Y12 receptor-implicated abnormal sympathoexcitatory reflex due to MI injury | The expression of lncRNA u.c48+ in SCGs was significantly upregulated in MI rats  Uc.48+shRNA improved abnormal changes in cardiac function and structure after MI  Clopidogrel (P2Y12 antagonist) improved abnormal changes in cardiac function and structure after MI |
| ***MOUSE*** |  |  |  |
| Ziegler et al | 2018 | To establish an animal model in mice that would allow primarily for denervation of the anterior ventricular wall. Upon validating its effectiveness in reducing myocardial sympathetic nerve density, we tested its effect in a mouse model of heart failure post-myocardial infarction | Bilateral SCGx caused the lower LV wall to be essentially devoid of any immunoreactivity for nerve endings compared to the base region of the anterior wall of sham-operated animals  Determination of plasma troponin T (cTnT) determined 24 h after LAD ligation were similar between mice subjected to MI alone compared to those with concomitant sympathetic denervation, indicating that the ischemic loss of cardiac myocytes was similar in both groups  Planimetric determination of infarct sizes in tissue sections stained with Sirius Red/Fast Green indicated medium- to large-sized infarcts in both MI groups. MI induced significant cardiac hypertrophy (as evidenced by an increase in heart weight-to-tibia length ratio) in control treated mice but not in mice subjected to sympathectomy  Quantitative analysis of cardiac myocyte dimensions in sections stained with the membrane stain WGA revealed a prominent and highly significant inhibition of MI-induced cardiac myocyte growth upon denervation  Local sympathetic denervation (SCGx) resulted in a drastic reduction of pro-NGF levels and reduced staining for TH  MI induced a prominent and long-lasting myocardial infiltration of inflammatory cells, the majority of which stained positive for CD68, identifying them as macrophages. SCG removal led to a significant decrease of these cells  The mRNA levels of the macrophage marker Cx3cr1 and the proinflammatory cytokine TNFα were reduced coinciding with an increase of the anti-inflammatory molecule Il-10 upon sympathetic denervation and LAD ligation  Fourteen days after the intervention echocardiography revealed significantly improved cardiac function of mice subjected to combined MI and SCGx compared with MI alone  LV strain imaging indicated better LV global myocardial function, with significant higher radial and longitudinal strain and radial displacement in mice subjected to MI and SCGx compared to MI alone |
| Ge et al | 2022 | To investigate the remodeling of the murine SCG as well as the bordering carotid body over time after MI | After MI, neuronal enlargement takes place; CHAT and TH are co-expressed in SCG neuronal cells  CHAT intensity is significantly downregulated 24h after MI in SCG; a negative correlation between neuronal cell size and relative CHAT expression was established in the SCG  Expression of neurotrophic factors BDNF and NGF was increased in the SCG after MI, concomitant with an increase in their TrK-receptor  An increased expression of GAP43 indicative of neuronal remodeling resulting in hyperinnervation after MI |
| Zhang | 2023 | To assess whether Schisandrin B can decrease the expression of P2X7 receptor in diabetic rats to protect the cardiovascular system | Pathological blood pressure, heart rate, heart rate variability, and sympathetic nerve discharge were ameliorated after administration of Schisandrin B. Moreover, the upregulated protein level of P2X7 receptor, NLRP3 inflammasomes, and interleukin-1β in diabetic rats were decreased after treatment, which indicates that Schisandrin B can alleviate the chronic inflammation caused by diabetes and decrease the expression levels of P2X7 via NLRP3. These findings suggest that Schisandrin B can be a potential therapeutical agent for DCAN. |

**Appendix F: Objectives and outcomes functional in vitro studies**

| **Functional – in vitro** | | | |
| --- | --- | --- | --- |
| **Author** | **Year** | **Objectives** | **Outcome** |
| ***RABBIT*** |  |  |  |
| Cheng et al | 2018 | To investigate the effects of ischemia and fluvastatin treatment on the ion channel characteristics of SCG neurons in a rabbit myocardial ischemia (MI) model. | The peak current densities of the MI-7d and MI-14d groups were significantly higher than that of the control group (n = 10, P < 0.05). By contrast, the peak current densities of the fluvastatin-7d and fluvastatin-14d groups were significantly reduced compared to that of the MI groups (n = 10, P<0.05).  The activation curves for IK of the five groups showed no significant statistical difference (n = 10, P>0.05). Fluvastatin and MI did not alter the activation characteristics of IK.  The peak current densities of the MI-7d and MI-14d groups were significantly increased compared with that of the control group. The I - V curves of the fluvastatin-7d and fluvastatin-14d groups were not significantly different from that of the control group (n = 10, P > 0.05). Thus, fluvastatin reversed the changes of the I- V curves of INa induced by MI.  The activation curves of the MI-7d and MI-14d groups were shifted toward the negative potential compared with that of the control group (n = 10, P < 0.05). The activation curves of the fluvastatin-7d and fluvastatin-14d groups were close to that of the control group (n = 10, P > 0.05). Thus, fluvastatin reversed the activation characteristics of INa induced by MI.  The ANOVA of the five inactivation curves showed no significant statistical difference (n = 10, P > 0.05) between these groups. Thus, fluvastatin treatment and MI did not change the inactivation characteristics of INa.  The recovery curves after the inactivation of the MI- 7d, MI-14d and fluvastatin-7d groups shifted toward the negative potential compared with the control group (n = 10, P < 0.05), and the recovery curves after the inactivation of the fluvastatin- 14d group were similar to control levels (n = 10, P > 0.05).  Therefore, the effects of fluvastatin on recovery curves after the inactivation of INa may be correlated with the duration of MI injury.  The calcium concentration in the MI-7d and MI-14d groups were increased slightly; however, there were no significant differences in the five groups (n = 10, P > 0.05).  Compared with the control group, the AP amplitude in the MI-7d and MI-14d groups were significantly higher (n = 10, P < 0.05), and APD90 levels were significantly shorter  (n = 10, P < 0.05). Fluvastatin treatment reversed AP amplitude and APD90 levels (n = 10, P > 0.05). Fluvastatin prevented the change of characteristics of AP induced  by MI and exerted protective effects on the electrical activity of SCG neurons. |
| Cheng et al | 2023 | To determine the changes in the ion channel characteristics of the SCG following myocardial infarction (MI) and the role of pretreatment with the P2Y12 receptor | P2Y12 receptor antagonist (TIC) pretreatment partly reversed P2Y12 expression and abnormal neuronal electrophysiological changes in SCGs after MI. |
| ***RAT*** |  |  |  |
| Furshpan et al | 1976 | To study whether in cultures with both adrenergic and cholinergic functions individual neurons are exclusively adrenergic or cholinergic or whether they can display both functions simultaneously | Some neurons inhibited, some excited and others first inhibited and then excited the cardiac myocytes  Application of drugs provided evidence for secretion of acetylcholine by the first group, catecholamines by the second and both acetylcholine and catecholamines by the third  Solitary neurons which inhibited the myocytes usually excited themselves at nicotinic synapses |
| Landis | 1976 | To study whether in cultures with both adrenergic and cholinergic functions individual neurons are exclusively adrenergic or cholinergic or whether they can display both functions simultaneously | Single neurons of either putative cholinergic or putative adrenergic character made morphological synapses on themselves (autapses)  Numerous axonal varicosities were present adjacent to the myocytes but no synaptic specializations were evident; 3) the endings of neurons which appeared to secrete catecholamines contained many small granular vesicles, while endings of neurons which appeared to secrete acetylcholine contained none, the endings of neurons which apparently secreted both catecholamines and acetylcholines contained only occasional small granular vesicles |
| Chun and Patterson | 1977 | To study the effect of NGF on the development of cholinergic sympathetic neurons | In the presence of rat heart cells the absolute requirement of neurons for exogenous NGF was partially spared  The ability of heart cells to support neuronal survival was due at least in part to production of a diffusible NGF-like substance into the medium  In mixed neuron-heart cell cultures, NGF increased both ACh and catecholamine production per neuron to the same extent |
| King et al | 1978 | To assess the effect of tyramine on the contraction rate of the co-cultured ventricular cells | The addition of 5 X 10 -6 M tyramine caused an increase in the contraction rate of heart muscle cells contacted by neuronal processes in 20 out of 23 neuron-muscle cell pairs examined. This increase in rate varied from 9 to 100%, the average being 48%. Heart cells cultured without neurons never responded positively to this concentration of tyramine |
| Schwab et al | 1981 | To examine the surface membranes of cultured SCG cells | The density of binding sites for concavalin A, ricin and wheat germ agglutinin increased with age in culture on both adrenergic and cholinergic cells  Soybean agglutinin increased on adrenergic axons, but failed to increase on cholinergic axons  Mature adrenergic neurons appeared to bind more WGA than neurons in CM cultures (cholinergic)  Tetanus toxin gold binding was uniform but low on axons of adrenergic and cholinergic neurons at all ages  Cholera toxin binding decreased with age on adrenergic axons; -->Growth in conditioned medium induces fundamental changes in the phenotype of developing sympathetic neurons involving the cell membrane as well as transmitter choice. Differences also appear with maturation. |
| De Ridder and De Potter | 1983 | To develop a new culture technique to allow immediate contact between the intact SCG and heart fragments avoiding substrate interaction | Histological analysis with light and electron microscopy revealed the interaction between the sympathetic ganglion and its target organ  Ingrowth of axons rich in neurofilaments and neurotubules and containing light and dense core vesicles, is observed  After 2 hr of contact on the semi solid medium all ganglia attach to the heart fragment  After 24 hr incubation on the shaker, microscopy reveals that the ganglia are arranged at one pole of the heart fragment but no clearcut border is seen between both compartments  After 3 and 6 days of confrontation the ganglia form one mass with the heart tissue. A layer of elongated fibroblastoid cells encircles the whole confrontation and the position of both compartments towardseach other has changed |
| Kessler et al | 1984 | To study the effects of neuron-target interactions on sympathetic, peptidergic expression, SP was examined in dissociated SCG cell cultures grown in association with target structures. | The association of SCG neurons with pineal (p=<0.005) or salivary gland (P<0.01) (normal SCG targets) resulted in a striking eightfold increase in peptide content, whereas SP failed to increase in neurons cultured with heart or gut |
| Lahtinen et al | 1986 | To determine the embryonic age at which the neurons of the SCG cocultured with atrium explants first grow fibres to the target tissue and to study the role of NGF in these interactions | The youngest stage at which the SCG showed increased nerve fibre growth was E15 (only with exogenous NGF or with E15 or P1 atrium explants), E14 resulted in enhanced fibre outgrowth only in the presence of atrium explants of E14 but not exogenous NGF or P1 atrium explants  The stimulation and orientation effects exerted by atrium explants of both P1 and E15 were statistically significant and did not differ from each other, this effect was totally abolished by anti-NGF in P1 and partially inhibited in E15, suggesting the stimulation of fibre growth is mediated by a coeffect of NGF and an unknown growth factor |
| Furshpan et al (1) | 1986 | To research transmitter status of individual sympathetic neonate-derived neurons during the transition from adrenergic to cholinergic status | Several lines of evidence indicate that the neuronally evoked hyperpolarizations described in this section were produced by ACh acting via muscarinic receptors on the myocytes  Moderate concentrations of atropine substantially or completely blocked the hyperpolarizations evoked either by the neuronally released agent or authentic ACh or bethanechol applied in quantities that roughly mimicked the neuronal effect  Many of the solitary neurons that produced this effect also produced a hexamethonium-sensitive excitation at autapses; thus, there was parallel evidence for secretion of ACh by the same neuron at another site  When fixed in permanganate the synaptic endings and varicosities made by such neurons possessed the SCV characteristic of choline& junctions in vivo  There is biochemical evidence for synthesis’ and storage of ACh by sympathetic neurons (neonate- or adult-derived) co- cultured with cardiac cells or grown in medium conditioned by such cells  In many microcultures, stimulation of the neuron evoked (as at least one of its effects) myocyte excitation that was blocked by adrenergic antagonists  When the myocytes were already beating, stimulation of such neurons caused an increase in beating rate; when the myocytes were not beating, stimulation evoked a depolarization in the myocytes and often initiated beating  Neurons functionally identified as apparently purely adrenergic were fixed in KMnO, and examined with the EM. All of the synaptic terminals and varicosities contained numerous small granular varicosities  In functionally cholinergic neurons small synaptic vesicles contained only electron-lucent centers, no small granular varicosities were observed  The mean proportions of small granular varicosities in the five adrenergic cultures were 59,67,72,76, and 85%.  A brief puff of perfusion fluid containing ACh and NE was applied to the microculture. The initial hyperpolarization was the response to ACh; the delayed excitation, the response to NE  In the presence of atenolol and phentolamine, a puff of ACh/NE evoked the hyperpolarization but not the onset of beating. The sensitivity of the myocytes to applied NE, and the block of both the NE response and the neuronally induced ex- citation by moderate concentrations of adrenergic antagonists, suggest that the neuron released NE. |
| Potter et al | 1986 | To study the neurotransmitter transition of a single neuron co-cultured with myocytes | Several dual function neurons were encountered that can synthesize both transmitters  The variation in the relative strengths of the adrenergic and cholinergic effects, from microculture to microculture, primarily reflected variations in transmitter status associated with the transition  The junctions and varicosities of dual-function neurons were found to contain a mixture of SGV, plausibly storage sites for NE, and small clear vesicles(SCV), plausibly storage sites for Ach  All the solitary neurons in which vesicle counts were made showed only single population of terminals. The implication is that cholinergic and adrenergic properties coexist within each terminal and that the synapses and varicosities are individually dual in status like the neuron as a whole.  There is heterogeneity in transition rates between the neurons. The direction of the transition is mostly unidirectional towards a (more) cholinergic status.  In multiple-neuron microcultures, the neurons were sometimes conspicuously different in status. providing further evidence for the heterogeneity in transition status.  about 78% (292) of neonate-derived neurons displayed at-least-cholinergic function. Only 8% (23) were apparently purely cholinergic (hatched bars). Among the at least- cholinergic cases were neurons that also displayed adrenergic, purinergic, or NAE functions. About 45% (13 1) of the at-least cholinergic neurons also exhibited adrenergic function; this is a lower limit, because for 95 of the at least-cholinergic group, the only test of function was the presence of neuron-neuron syn-apses; probably most of the 95 neurons were also adrenergic cholinergic in status. If these 95 neurons are excluded, 66% of the remaining 197 neurons were (at least) adrenergic/cholinergic in status.  About 77% of the 214 neurons were at-least-adrenergic, the proportion of neurons that were detectably at-least-adrenergic also fell during the second through the seventh week  Of the 81 adult derived neurons assayed in microcultures, all but 5 expressed at- least-adrenergic function.  The incidence of cholinergic function was considerably lower during the weeks 2-7 in culture for adult-derived than for neonate-derived neurons.  Seven neonate-derived neurons grown in medium that contained 3.1 PM TTX and 1 mM hexamethonium were examined, these concentrations completely block action potentials and neuron-neuron synapse. The block did not prevent formation of effective junctions or (assuming adrenergic status at the outset) transmitter plasticity, at least in neonate-derived neurons. |
| Furshpan et al (2) | 1986 | To study the neuron-evoked hyperpolarizations that were completely insensitive to atropine | Several lines of evidence are consistent with the idea that the atropine-resistant hyperpolarizations described here were mediated, at least in large part, by adenosine  The neuronally evoked effect was strongly or completely blocked by methylxanthines at concentrations reported to block adenosine receptors in other preparations  A specific degradative enzyme, adenosine deaminase, was also an effective antagonist  These agents exhibited specificity of action to the extent that they failed to block adrenergic and cholinergic responses  Puffed adenosine mimicked the neuronally evoked response in the 1-10 µM concentration range; the response to puffed adenosine was strongly or completely blocked by the methylxanthines or adenosine deaminase at the same concentrations that affected the neuronally evoked response |
| Matsumoto et al | 1987 | To illustrate the nonadrenergic excitatory effects of sympathetic neurons on myocytes  To illustrate the impressive diversity of transmitter states expressed by the microcultured neurons | The neonate-derived neuron exerts a nonadrenergic excitatory effect on the myocytes  Some microcultures showed secretion of serotonin  The nonadrenergic excitatory effect was diminished or abolished by serotonin blockers or reserpine  In some cases, an unidentified agent X also produced nonadrenergic excitation |
| Conforti et al | 1991 | To gain a better understanding of the influence of the sympathetic innervation on the AP characteristics of the cardiomyocytes and of the synaptic process in co-culture | Prolonged stimulation (5-10 s duration) increased the frequency of APs in the latent pacemaker myocytes, suggestion the formation of functional synapses between these myocytes and the neurons  The mean latency from the first neural AP in the train to the beginning of the myocyte depolarization was 2.6 ± 1.2 s. The mean peak altitude, duration (measured at half Ampl.) and rise time (measured from from the beginning of the depolarization to the peak amplitude) were 8.0 ± 1.8 mV, 1.34 ± 0,37s and 0,76± 0,15 s, respectively  Elicitation of a single or a train of APs in the neuron induced a slow EPSP in the myocytes. The RP of the cells where slow EPSPs were recorded was -72 ± 2 mV (n=6). This RP is significantly lower then that observed in regularly beating myocytes (P<0.05)  After nifedipine treatment, stimulation of the neuron induced a slow EPSP in the impaled myocyte. No significant difference was observed with or without treatment. |
| Kannan et al | 1994 | To research the influence of lymphoid tissues on neurite outgrowth | At 24 hr: neurite outgrowth toward heart (52.9, 7.40; P<.05) was significantly greater than toward Spinal Cord. In addition, neurite outgrowth towards the heart was significantly greater than away from the heart (52.9, 7.40 vs. 34.2, 6.73; P < .001)  At 48hr: Neurite outgrowth toward heart (65.3, 6.61) was significantly (P < .050) greater than towards SC (21.5, 3.52). The neurite outgrowth away from heart was also dramatically increased at 48 hr compared with 24 hr(61.9, 7.69 vs. 34.2, 6.73)  It was shown that the rank order of the capacity to induce neurite outgrowth from SCG was heart 5: thymus > spleen > MLN 2 SC explants. When SCG were co-cultured with SC, heart, and thymus explants for 24 hr (Fig. 4), the relative effects seen in Series 1 were confirmed and the overall difference was statistically significant by ANOVA (F[2,21] = 3.54; P < .050), although differences between each pair of targets were not detected by Tukey's tests  The addition of 10 pg/ml of anti-NGF completely blocked the outgrowth of neurites from SCG in the presence of both 1 and 10 ng/ml NGF. The addition of anti-NGF to the co-cultures of SCG and heart clearly blocked the neurite outgrowth. |
| Lockhart et al | 1997 | To demonstrate that NGF can also potentiate synaptic transmission between sympathetic neurons and cardiac myocytes in vitro  To present evidence that synaptic potentiation works through a synaptic mechanism  To demonstrate that NGF produces a long-term enhancement of synaptic transmission in cultures of sympathetic neurons and cardiac myocytes | In addition to its role as a survival factor, NGF plays both acute and long-term roles in the regulation of developing sympathetic synapses in the cardiac system  The beat rate of cardiac myocytes increased during stimulation of a connected neuron  NGF produced a pronounced, reversible enhancement of synaptic strength  TrkA is expressed primarily by neurons in cultures with NGF  NGF did not alter the response of myocytes to application of NE  The concentration of NGF in the growth medium affects the level of synaptic transmission in the co-cultures |
| Ulupinar et al | 1998 | To assay growth vigor and patterns of neurites from different peripheral ganglia in the whisker pad, forepaw and heart explants | Vigorous innervation from SCG into the heart |
| Hasan et al | 2006 | To investigate whether NGF produced by peri-infarct cells induces sympathetic outgrowth. | Neurite outgrowth from sympathetic ganglia was significantly greater at post-ligation days 7–14 as compared to control tissues. |
| Li et al | 2010 | To examine whether myocardial infarction will induce changes in systolic blood pressure, heart rate and respiration, the expression value of TH and P2X2/3 receptor in SCG and myocardial tissues, and the effects of P2X2/3 receptor antagonist A-317491 on above changes | The amplitude of the currents in myocardia ischemic group was much larger than those obtained in control group after administration of ATP with the same concentration and the mean peak was 2.1 times higher than that measured in control rats treated with 100 mMATP  The amplitude of the currents in myocardial ischemic rats treated with A-317491 group was lower than that in myocardial ischemic rats and no difference was observed when compared to control |
| Miwa et al | 2010 | To investigate the effect of neurotrophic factors on sympathetic neurite growth towards cardiomyocytes | NGF, BDNF, GDNF significantly increased the density of neurites  GDNF enhanced neurite outgrowth even under the NGF depleted culture condition and no difference in the amount of soluble NGF between the cultures with and without BNDF, excluding an indirect effect of GDNF via NGF |
| Takeuchi et al | 2011 | To develop a new technique for co-culture of sympathetic ganglion neurons and cardiomyocytes using microfabrication  To evaluate the functional relationship of the two components in terms of changes in the beat rate of the cardiomyocytes after applying electrical stimulation to the sympathetic neurons | The pulse frequency had a significant effect on the beat rate ratio, as did the interaction between the number of pulses and their frequency  Bonferroni’s multiple comparison post hoc test demonstrated a significant difference between 1 Hz and 10 Hz at 600 pulses  The beat rate of the VMs after electrical stimulation did not increase significantly when propranolol was added to the culture medium (P < 0.01, paired t test) |
| Kong et al | 2013 | This study aimed to define the role of P2X7 receptor of the superior cervical ganglion neurons in rat myocardial ischemic injury and explore the mechanism of cellular signal transduction after activation of P2X7 receptor | Majority of the SCG neurons in control group (90%, 63/70) and myocardial ischemic group (93.7%, 75/80) responded to the external application of BzATP (1–1000 lM). Amplitudes of the currents activated by P2X7 receptor agonist at the same concentration in SCG neurons of myocardial ischemic rats were much larger than those in control rats.  The maximal response of IBzATP in neurons of myocardial ischemic rats was higher than that in control rats. The EC50 values in control group and myocardial ischemic group were around 82.80 lM and 54.30 lM, respectively. The dose–response curve for BzATPactivated currents in SCG neurons was shifted upward markedly after myocardial ischemic injury  Effect of BBG (50 nM) on BzATP (100 lM)-activated currents in myocardial ischemic rats was strengthened in comparison with that in the control rats  A dose-dependent inhibiting effectof BBG (1–100 nM) on BzATP-activated currents was observed in control and myocardial ischemic group. At the same concentrations, the inhibitory effect of BBG in myocardial ischemic rats was stronger than that in control rats (p < 0.05)  The amplitudes of IBzATP were potentiated obviously after myocardial ischemic injury at all HP. The I–V curve was shifted to the right in the range of negative potential compared with that in the control group  The inhibiting effect of BBG on BzATPactivated currents was 42.35 ± 8.36% (n = 8) in normal intracellular liquid (p < 0.05) (Fig. 5). The inhibiting effect of BBG on BzATPactivated currents was 28.42 ± 6.27% (n = 8) in intracellular liquid of containing GF109203X. The inhibiting effect of BBG on BzATP- activated currents in intracellular liquid of containing GF109203X was obviously lower than that in normal intracellular liquid (p < 0.01)  The inhibiting rang of BBG on BzATP-activated currents in intracellular liquid of containing GF109203X was obviously lower than that in normal intracellular liquid (p < 0.05). BzATP-activated currents in intracellular liquid of containing GF109203X (10 lM) were small enhanced to 10.75 ± 2.66 (n = 8) in comparison with those in normal intracellular liquid (p > 0.05) Thus PKC inhibitor GF109203X could relive the inhibiting effect of BBG on BzATP-activated currents. |
| Miwa et al | 2013 | To examine the potency of GDNF and NGF in stimulating sympathetic axon growth and enhancing functional coupling between sympathetic neurons and ventricular myocytes  To show the potent action of endogeneous GDNF for sympathetic axon guidance using ventricular myocytes overexpressing GDNF | Fractions of axons and synapsin-I-positive area over the surface of ventricular myocytes were markedly increased with GDNF  Pre- and postsynaptic stimulation of beta1-adrenergic receptors with nicotine and noradrenaline resulted in an increase of the spontaneous beating rate of ventricular myocytes co-cultured with sympathetic neurons in the presence of GNDF  GDNF overexpressing ventricular myocytes by adenovirus vector attracted more axons from sympathetic neurons compared with mock-transfected ventricular myocytes |
| Liu et al | 2014 | To examine the effects of puerarin on the sympathoexcitatory response induced by myocardial inschemia and explore the relationship with P2X3 receptor in rat SCG | The inhibitory effect of puerarin (10uM) on ATP(100uM)-activated currents was higher in MI than control  The transient ATP current can be inhibited by A317491  Puerarin dose-dependently (1-100uM) inhibited ATP-activated currents in control and MI  At the same concentrations, the inhibitory effect of puerarin was stronger in MI than control |
| ***MOUSE*** |  |  |  |
| Coughlin et al | 1981 | To isolate a fraction from mouse heart-cell-conditioned medium to stimulate SCG development  To establish and maintain cell cultures of heart | A fraction of medium conditioned by embryonic mouse heart cells in culture stimulates neurite outgrowth, elevates specific activities of tyrosine hydroxylase and choline acetyltransferase in SCG explants and enhances survival of dissociated sympathetic neurons in culture  The conditioned medium fraction is insensitive to anti-NGF antiserum |
| Coughlin and Kessler | 1982 | To characterize the molecular properties of conditioned medium factor and examine its biological activity | Anti-HCM factor inhibited NGF-stimulated neurite outgrowth from the neonatal SCG  Anti-HCM factor did not inhibit the NGF-stimulated increase in TH activity  SCG incubated for 3 days in the presence of anti-HCM factor were capable of producing neurites when washed and cultured in medium free of antiserum |
| Rawdon and Dockray | 1983 | To examine whether or not s1/s1 mice are deficient in the production of trophic factors needed for one class of neural crest derivative, i.e. sympathetic neurones  To assess to what extent sympathetic ganglia in s1/s2 mice suffer from a failure to respond adequately to trophic factors; To examine the consequences of absence of intrinsic neurones in the distal colon of s1/s1 mice on the production of trophic factor by this region | Directional outgrowth of neurites from SCG towards atrium was observed in both normal and s1/s1 mice |
| Uchida and Tomonaga | 1985 | To examine whether dissociated aged sympathetic neurons respond to NGF and HCM in culture | Neurite production of early adult neurons was enhanced by NGF, HCM-S (HCM in solution) or P-HCM (HCM in polyornithine binding condition)  Neurite production of aged neurons was enhanced only by HCM-S  HCM-S did not promote neurite elongation in neurons at any age  Neurite elongation of early adult neurons was enhanced by NGF or P-HCM  Neurite elongation of aged neurons was enhanced by P-HCM |
| Rawdon | 1991 | To establish when target organs first produce neuronotrophic factors  To establish to what extent such activity can be related to NGF and other neuronotrophic substances | The atrium produces neuronotrophic factors to SCG from E11, most marked towards E17 and P1  NGF is the predominating neuronotrophic factor in the atrium |
| Shcherbakova et al | 2007 | To analyze the organization of signaling molecules at the site of innervation of cardiac myocytes by sympathetic neurons | Sympathetic neurons form functional synapses with neonatal cardiac myocytes in culture  The myocyte membrane develops into specialized zones that surround contacting axons and contain accumulations of the scaffold proteins SAP97 and AKAP79/150 but are deficient in caveolin-3  The β1ARs are enriched within these zones, whereas β2ARs are excluded from them after stimulation of neuronal activity |
| Ge et al | 2020 | To investigate whether mesenchymal EPDCs influence cardiac sympathetic innervation. | Mesenchymal EPDCs promote sympathetic neurite sprouting is via paracrine signaling, and indicate a role for NGF, Endothelin-1 and SEMA3A in the process. |
